# Compositional and Functional Changes in Microbial Communities of Composts Due to the Composting-Related Factors and the Presence of Listeria monocytogenes

**DOI:** 10.1128/spectrum.01845-21

**Published:** 2022-06-15

**Authors:** Hongye Wang, Vijay Shankar, Xiuping Jiang

**Affiliations:** a Department of Food, Nutrition, and Packaging Sciences, Clemson University, Clemson, South Carolina, USA; b Center for Human Genetics, Clemson University, Greenwood, South Carolina, USA; Tufts University

**Keywords:** compost, microbial community, *L. monocytogenes*, competitive exclusion microorganisms, 16S targeted sequencing, metagenomic sequencing, metatranscriptomic sequencing, *Listeria monocytogenes*

## Abstract

Listeria monocytogenes is a leading foodborne pathogen that can contaminate fresh produce in farm environment, resulting in deadly outbreaks. Composts contain a diversity of microorganisms, and some of them may be compost-adapted competitive exclusion microorganisms against L. monocytogenes. To understand interactions between compost microflora and the pathogen, both dairy- and poultry-wastes based composts (*n* = 12) were inoculated with L. monocytogenes, and then analyzed by next-generation sequencing approaches along with culturing methods. DNA extraction and enumeration of L. monocytogenes were performed at 0 and 72 h post-incubation at room temperature. The major bacterial phyla were identified as *Firmicutes* (23%), *Proteobacteria* (23%), *Actinobacteria* (19%), *Chloroflexi* (13%), *Bacteroidetes* (12%), *Gemmatimonadetes* (2%), and *Acidobacteria* (2%). The top three indicator genera enriched in different compost types were identified by LEfSe with LDA score > 2. The interactions between L. monocytogenes and indigenous microflora were limited as no significant changes in the dominant microbial members in compost ecosystem, but some discriminatory species such as *Bacillus*, *Geobacillus*, and *Brevibacterium* were identified by Random Forest analysis. Besides, changes in metabolic pathways and the increased abundance of bacteriocins category in the compost samples containing L. monocytogenes after 72 h postinoculation were revealed by metatranscriptomic sequencing. Taken together, the compost-related factors such as compost types, composting stages, and the collection farms are major drivers that affect compost microbial compositions, and the analysis of compost metagenome implied that interactions between L. monocytogenes and compost microflora may include competition for nutrients and the presence of antimicrobials.

**IMPORTANCE** Listeria monocytogenes has been recognized as the etiological agent causing foodborne disease outbreaks, with fresh produce as vulnerable for contamination at even preharvest stage. Owing to the richness in microbial community, compost may mediate suppression of pathogens. In this study, the impact of compost-related factors and L. monocytogenes intrusion on dynamic changes in compost microbiome was investigated by next generation sequencing techniques. The compost-related factors such as compost types, composting stages, and the collection farms are major drivers that affect compost microbiome. The interactions between L. monocytogenes and compost microflora may include the competition for nutrients and the presence of antimicrobials produced by native compost microorganisms as potential competitive exclusion microorganisms. Findings from this study are important for the composting industry to understand the composition and functionality of microbial community in their products and help developing organic fertilizers fortified with anti-L. monocytogenes competitive exclusion microorganisms.

## INTRODUCTION

Listeria monocytogenes is ubiquitous in nature, including soils and animal wastes. As a leading foodborne pathogen, L. monocytogenes poses a major public health risk due to its ability to thrive in both farming and food processing environments and the severity of listeriosis in the susceptible populations (1, 2). The improperly treated biological soil amendments, such as animal waste-based composts, can introduce L. monocytogenes to fresh produce during growing season (3). The prevalence of L. monocytogenes in animal waste or associated produce field can be as high as 50% as reported in different locations (2, 4, 5).

A key to develop mitigation strategies against this pathogen is to understand its ecology. Animal wastes used in compost production are considered as a nutrient-rich and complex ecosystem that contains diverse groups of microorganisms spanning from prokaryotes to eukaryotes. In addition to the generation of moderate levels of heat during the composting process, compost microbial community may also carry out suppressive activities against a variety of plant and human pathogens (6–8). These beneficial microorganisms with competitive exclusion activities can be utilized as environmentally friendly biological control agents to reduce the pathogen contamination (9, 10). Additionally, many studies have concluded that microbial diversity is a key factor in reducing pathogen outbreaks (11, 12). As such, fully understanding the microbial interactions in compost ecosystem by advanced approaches can assist with the discovery of potential competitive exclusion (CE) microorganisms from animal waste-based composts.

Genomic techniques based on PCR amplification of the conserved and variable regions of the microbial genome (16S rRNA gene) allow direct sequencing of these PCR amplicons using high-throughput next-generation sequencing. The 16S rRNA gene sequencing has been used to characterize the microbial community structure in compost samples, with *Proteobacteria*, *Bacteroidetes*, *Firmicutes*, *Actinobacteria*, and *Chloroflexi* being identified as the predominant bacterial members in dairy waste-based compost (13–16). Besides, dynamic changes in the bacterial and fungal communities during cow manure and corn straw composting process were studied. Results showed that *Actinomycetales* and *Sordariomycetes* are the indicators of bacteria and fungi in the compost maturation phase, respectively, whereas *Steroidobacter* and *Actinomadura* (bacteria) and *Coprinus* (fungi) dominate in the active thermophilic phase (17). Moreover, the feed of agricultural animal can affect the microbial composition in their composted manure (16).

With the studies performed to investigate the microbial composition during composting under field conditions, there is a lack of information to what extent the source of animal waste and other composting related factors affect the microbial composition in commercially available animal waste-based composts. Therefore, a practical understanding of microbial compositions in animal waste-based composts sold in US is needed. Additionally, most sequencing results were only based on the sequencing of genomic DNA extracted from compost samples without any pretreatment. A major limitation of genomic DNA sequencing analysis is the inability to differentiate live (dormant cells as well as growing or non-growing but metabolically active cells) and dead cells. To avoid the extraction of DNA from dead cells, investigators used a pretreatment viability assay with propidium monoazide (PMA) before DNA extraction (18). Therefore, DNA-based sequencing combined with PMA treatment can theoretically analyze DNA from living cells only, which could serve as a better approach to study the active microbial members in animal waste-based compost.

Nonetheless, microbial community based on 16S rRNA gene sequencing has its own limitations, such as only being able to infer functional potential through prediction which is highly based on how complete the databases of whole genomes are (19). On the other hand, analyzing the metagenome can add additional taxonomic and functional signature to community profiling by addressing all the different types of changes stimulated by pathogen inoculation. Furthermore, analyzing the metatranscriptome is especially useful in examining the changes of functional genes in compost, which can be impacted and altered by a diverse array of transitory factors, such as moisture, temperature, microbial species, and composting stage or ingredients. To our knowledge, despite a few metagenomics studies of thermophilic composting processes, there is a paucity of data and in-depth investigations using high throughput-sequencing to examine the microbiota of commercial animal waste-based compost products, albeit on investigating the human pathogen interactions with indigenous microorganisms in animal waste-based biological soil amendments using functional metatranscriptomic sequencing (14).

To fill these research gaps, we analyzed the microbial community of commercial compost products with and without L. monocytogenes using next-generation sequencing approaches. The objective of this study was to identify the composition and functional capabilities of compost microbiome in a variety of composts as affected by different factors, including compost types, composting stage, collection farms, moisture contents, and the inoculation of L. monocytogenes. Ultimately, findings from this study may assist with in-depth understanding of compost microbiome and lead to further research on discovering compost-adapted CE microorganisms that could be potentially added to the biological soil amendment to control L. monocytogenes.

## RESULTS

In this study, both dairy wastes- and poultry wastes-based composts (*n* = 12) either in active or finished stage were collected from composting facilities across the U.S. DNA extraction and enumeration of L. monocytogenes in the compost samples adjusted to 40 or 80% moisture were performed at 0 and 72 h post-incubation at room temperature. Then the microbial community structures and functional genes profiles in a variety of animal waste-based compost samples spiked with or without L. monocytogenes were analyzed by next-generation sequencing approaches.

### Compost analysis and survival of spiked L. monocytogenes.

Chemical characteristics of poultry and dairy compost samples collected from 6 farms were shown in Table S1 in the supplemental material. For the received dairy composts, both active and finished compost samples collected from dairy farm #3 had relatively higher carbon to nitrogen ratio (C:N), organic matter, and moisture contents compared to the compost samples collected from the other two farms (*P < *0.05). For the received poultry compost, the lowest (*P < *0.05) organic matter was observed in the compost samples collected from poultry farm #3. The population of total cultivable aerobic bacteria, heterotrophs, thermophiles, *Enterobacteriaceae*, yeast/mold, and actinomycetes in the collected compost samples ranged from approximately 6.8 to 9.7, 5.6 to 8.9, 3.3 to 8.6, <2.1 to 6.1, <2.1 to 6.3, and 5.7 to 8.7 log CFU/g, respectively (Table S2 in the supplemental material). For all categories, relatively higher levels of total aerobic bacteria, heterotrophs (except for poultry farm #3), and thermophiles were observed for the active compost compared to the finished compost collected from the same farm. The population level of yeast/mold was found to be significantly (*P < *0.05) lower than the population of culturable bacterial species. After selective enrichment, there were some black colonies observed on Oxford agar, but those black colonies were confirmed as non-L. monocytogenes with PCR targeting the *hlyA* gene. The reductions of spiked L. monocytogenes in compost at room temperature after 72 h of incubation ranged from 0.1 to 1.1 log CFU/g, but regrowth occurred in a few samples with 80% moisture (Table 1).

**TABLE 1:** Population change of L. monocytogenes in biological soil amendments after 72 h of incubation at room temperature^*a*^

Farms	Compost type	Population changes of *L. monocytogenes* (log CFU/g) in the compost with different MC^*b*^	
		40% MC	80% MC
Dairy farm #1	Active	0.1 ± 0	0.7 ± 0.2
	Finished	0.1 ± 0.2	0.5 ± 0.4
Dairy farm #2	Active	−0.3 ± 0.3	−0.4 ± 0.2
	Finished	−0.6 ± 0.2	−0.7 ± 0.1
Dairy farm #3	Active	0.3 ± 0.1	−0.3 ± 0.3
	Finished	−1.1 ± 0.2	−0.8 ± 0.2
Poultry farm #1	Active	−0.4 ± 0.1	−0.1 ± 0.2
	Finished	−0.5 ± 0.2	−0.8 ± 0.1
Poultry farm #2	Active	−0.1 ± 0.3	0.5 ± 0.1
	Finished	−0.6 ± 0.3	1.5 ± 0.2
Poultry farm #3	Active	−0.3 ± 0.2	−0.4 ± 0.1
	Finished	0.1 ± 0	−0.8 ± 0.3

a 0.5 log change was considered as significantly growth/decrease based on the data obtained for all compost samples.

b MC = moisture content.

### Core microbiome and biomarkers of dairy and poultry composts.

**FIG 1:**
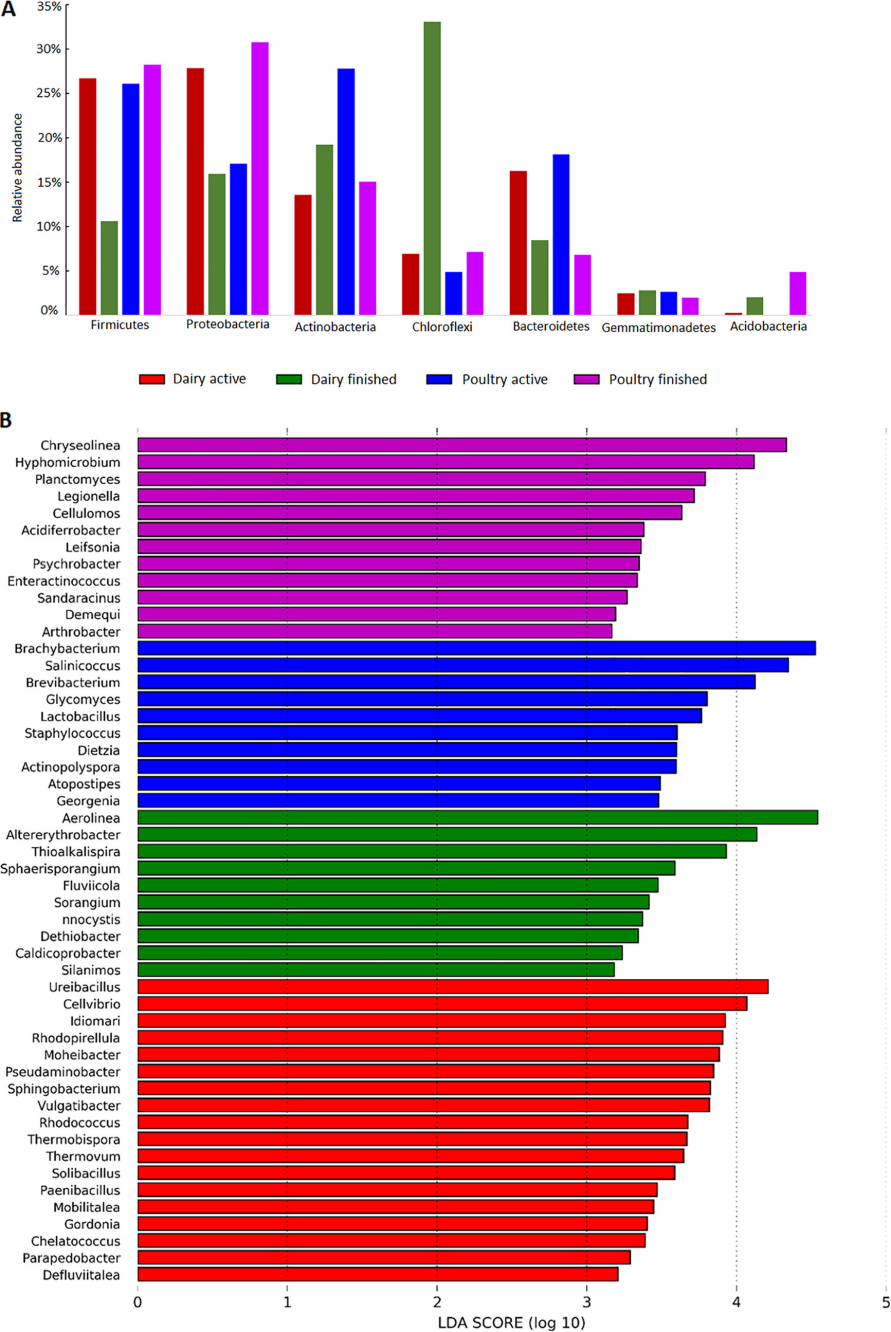
Relative abundance of the most common phyla (i.e., each representing >1% of total reads) present in four different compost types (dairy active compost, dairy finished compost, poultry active compost, and poultry finished compost) (A); LEfSe’s output of differentially abundant bacterial taxa among four compost types (B). Significant bacterial genera were determined by Kruskal-Wallis test (*P < *0.05) with LDA score greater than 2.

The average number of chimera-free sequences for each compost type was 55,420 ± 18,610 for further analysis (Table S3 in the supplemental material). The taxonomic composition of four types (active dairy, finished dairy, active poultry, and finished poultry) of compost samples was examined without L. monocytogenes inoculation to reveal the indigenous microbial communities of compost. Overall, the major bacterial phyla observed in all farms were *Firmicutes* (23%), *Proteobacteria* (23%), *Actinobacteria* (19%), *Chloroflexi* (13%), *Bacteroidetes* (12%), *Gemmatimonadetes* (2%) and *Acidobacteria* (2%), accounting for 94% of relative abundances in all compost samples. At phyla level, *Chloroflexi* represented a significantly greater proportion of relative abundances in finished dairy compost, followed by *Proteobacteria* and *Firmicutes* in finished poultry compost, *Actinobacteria* and *Firmicutes* in active poultry compost, and *Proteobacteria* and *Firmicutes* in active dairy compost (Fig. 1A).

At the genus level, Linear Discriminate (LEfSe) was used to detect microbial genera that differed significantly among compost types, with LDA score as >2 was considered significant biomarkers for that compost type. As indicated by LEfSe’s output (Fig. 1B), the top 3 indicator genera (biomarkers) enriched in finished poultry, active poultry, finished dairy, and active dairy composts were *Chryseolinea*, *Hyphomicrobium* and *Planctomyces*; *Brachybacterium*, *Salinicoccus* and *Brevibacterium*; *Aerolinea*, *Altererythrobacter* and *Thioalkalispira*; and *Ureibacillus*, *Cellvibrio* and *Idiomari*, respectively. As for other experimental factors, clear distinction in dominant bacterial genera between the compost samples with 40 and 80% moisture contents was visible, and this difference was more pronounced after 72 h of incubation (Fig. S1 in the supplemental material). However, the influences on the compost microbial composition from the spiked L. monocytogenes was not as strong (*P > *0.05%) as that from moisture content or incubation length.

### Compost type, moisture content and incubation time significantly affect diversity.

**FIG 2:**
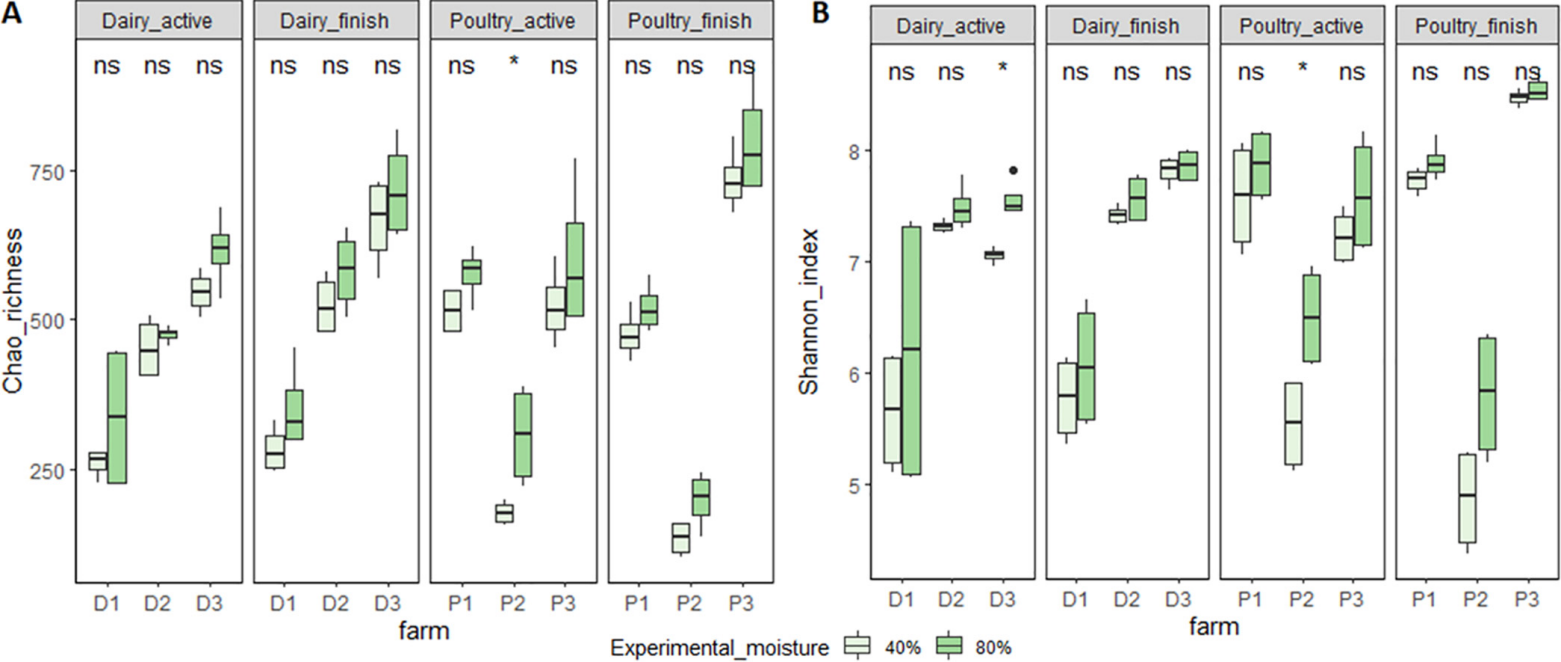
Effect of compost type on alpha diversity, chao richness (A) and Shannon_index (B), was tested using Kruskal-Wallis analysis, and the significant levels with Benjamini–Hochberg FDR adjusted *P*-value were added on the plots. P1 = poultry farm 1, P2 = poultry farm 2, P3 = poultry farm 3; D1 = dairy farm 1, D2 = dairy farm 2, D3 = dairy farm 3. The effect from moisture contents on alpha diversity was tested using Wilcoxon signed-rank test, and the significant levels were added on the boxplot (ns, not significant difference with *P*-value > 0.05; *significant difference with *P-*value < 0.05).

Chao richness and Shannon indices were used to quantify microbial species richness and diversity in compost samples. Results from Wilcoxon signed-rank test using experimental variables on all compost samples were shown in Table 2. Among the experimental variables, α-diversity indices were significantly affected by moisture content and incubation time, but not by *Listeria* inoculation (Table 2). On the other hand, the species diversity and richness differed significantly [Benjamini–Hochberg False Discovery Rate adjusted *P-*value (BH-FDR) adjusted *P*-values < 0.05] among the compost types as indicated by the non-parametric Kruskal-Wallis test (Fig. 2). The finished compost from poultry farm #2 that had the lower diversities (Fig. 2) had the regrowth (1.5 log CFU/g) of spiked *L monocytogenes* at higher moisture content (Table 1).

**TABLE 2:** Wilcoxon signed-rank test for experimental variables that affects the alpha diversities for compost sample overall^*a*^

Experimental variables	Chao_richness	Shannon
*Listeria* inoculation	0.612	0.8385
Moisture contents	0.015* ^*b*^	0.0034**
Incubation time	0.000006***	0.000000576***

a Paired test for incubation time, unpaired for other two factors; Significant level was indicated by Benjamini-Hochberg FDR adjusted *P*-value, with *, P <* *0.05, **, P <* *0.01, ***, P <* *0.001.

b The bold numbers are indicating the significant levels (BH-FDR adjusted *P* value) which are less than 0.05.

### Microbial community composition and response to environmental variables are farm specific.

**FIG 3:**
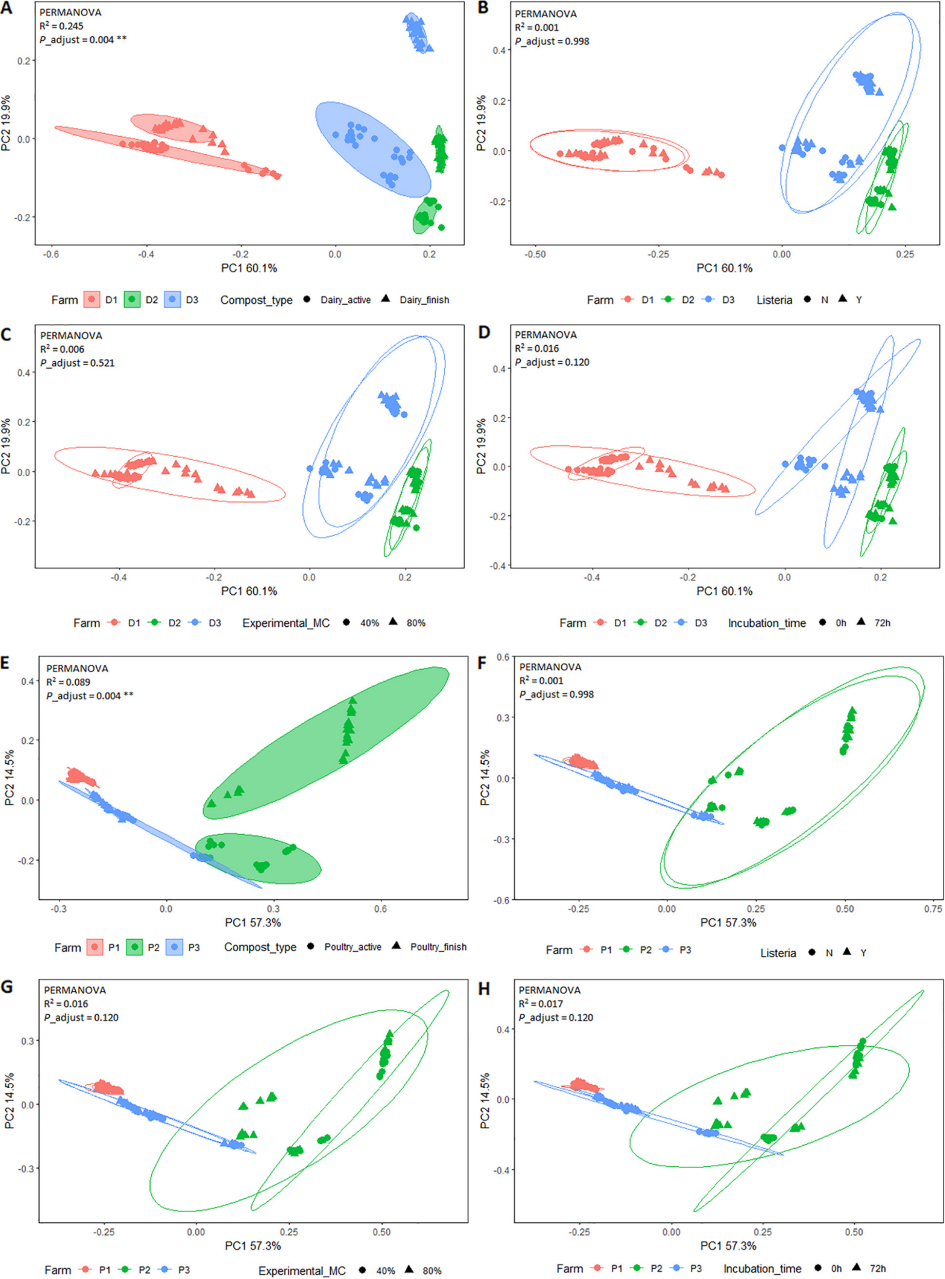
Weighted UniFrac distance-based principal-coordinate analysis (PCoA) plots for dairy (A, B, C, D) and poultry compost (E, F, G, H). P1 = poultry farm 1, P2 = poultry farm 2, P3 = poultry farm 3; D1 = dairy farm 1, D2 = dairy farm 2, D3 = dairy farm 3. Shaded polygons (A and E) were applied to compost samples collected from the same stage, and transparent polygons were applied to the compost samples with different treatments, including L. monocytogenes inoculation (B and F); experimental moisture contents (C and G), and incubation time (D and H). The variables in each PCoA plot were indicated by the legend under each panel.

The distances from the β-diversity of microbial communities for each farm were visualized in the exploratory principle-coordinate analysis (PCoA) plot (Fig. 3). Sample relationship matrix was calculated using a weighted UniFrac distance, which incorporates phylogenetic relatedness among 16S rRNA gene sequences when calculating distance. The PCoA plots showed that the microbial composition of compost samples collected from each farm formed a distinct cluster, while for each farm, microbial composition of active compost samples appeared to be more distinct from those of finished compost (Fig. 3A and E). Overall, for all farms, the microbial compositions of compost samples were not separated by the experimental factors (moisture contents, incubation length, and the presence of L. monocytogenes; Fig. 3B–D, F–H). This observation was consistent among samples collected from dairy and poultry farms. In line with the PCoA ordination, Permutational multivariate analysis of variance (PERMANOVA) showed that the composition of the microbiota varied significantly by composting farm with BH-FDR adjusted *P*-values < 0.05 (Fig. 3A and E). However, all the experimental factors had no significant effects on the compositions of microbiota with BH-FDR adjusted *P*-values > 0.05.

In addition, the effects of experimental variables on microbial composition within each farm were studied by canonical correspondence analysis (CCA). Three experimental conditions (moisture, incubation length, and presence of L. monocytogenes) were used as constraining variables. The CCA test was performed within each farm including both active and finished compost samples. The variation in microbial compositions of compost samples from dairy farms #1 and #3, and poultry farm #2 were significantly associated with both incubation time and experimental moisture (BH-FDR adjusted *P < *0.05), whereas the microbial composition of compost samples from poultry farm #3 was only affected by incubation time (BH-FDR adjusted *P < *0.05) (Table 3). It is noteworthy that the total dissimilarity due to L. monocytogenes was a small percentage of the overall variations, which could be inferred from χ^2^ residual that was calculated from the CCA models (Table 3).

**TABLE 3:** The effects of experimental variables on microbial composition within each farm as revealed by canonical correspondence analysis

Farms	exptl factors	F	*P*-values^*a*^	χ^2^
Dairy farm #1	**exptl moisture**	**4.8637** ^*b*^	0.005**	**0.16077**
	**Incubation time**	**6.2962**	0.005**	**0.20812**
	*L. monocytogenes* inoculation	0.6487	0.917	0.02144
	Residual			1.45439
Dairy farm #2	exptl moisture	1.1631	0.363	0.04667
	Incubation time	1.8146	0.169	0.07282
	*L. monocytogenes* inoculation	0.6486	0.917	0.02603
	Residual			1.76564
Dairy farm #3	**exptl moisture**	**2.8826**	0.033*	**0.11812**
	**Incubation time**	**2.9677**	0.041*	**0.12161**
	*L. monocytogenes* inoculation	0.5889	0.917	0.02413
	Residual			1.80296
Poultry farm #1	exptl moisture	1.2030	0.345	0.06119
	Incubation time	2.3772	0.113	0.12091
	*L. monocytogenes* inoculation	0.5597	0.917	0.02847
	Residual			2.23804
Poultry farm #2	**exptl moisture**	**6.3641**	0.005**	**0.23171**
	**Incubation time**	**9.1743**	0.005**	**0.33403**
	*L. monocytogenes* inoculation	0.5080	0.917	0.01850
	Residual			1.60201
Poultry farm #3	exptl moisture	1.5417	0.169	0.13613
	**Incubation time**	**2.7471**	0.022*	**0.13613**
	*L. monocytogenes* inoculation	0.6603	0.917	0.03272
	Residual			2.18045

a Significant level was indicated by Benjamini-Hochberg FDR adjusted *P*-value, with *, P <* *0.05, **, P <* *0.01, ***, P <* *0.001. Residual and F-statistics were calculated from the CCA models.

b The bold numbers are indicating the significant levels (BH-FDR adjusted *P*-value) which are less than 0.05.

### Discriminant microbial members that classify compost samples into L. monocytogenes inoculated and uninoculated samples.

Microbial members that separate inoculated and uninoculated communities in each farm were identified by Random Forest (RF) machine learning discriminant analysis. Based on the error rates generated by the RF function, the overall accuracy rates for dairy and poultry farms were 75% and 70%, respectively. Considering the complexity of microbial composition in compost samples, the entries from all samples with total reads <50 were excluded while performing RF analysis for each farm. Figure S2 shows the top 20 discriminatory features respectively in dairy and poultry composts collected in each farm ordered by the Mean Decrease Accuracy (MDA) and the Mean Decrease in Gini (MDG). Both MDA and MDG are used for ranking and selecting the important features that classify the L. monocytogenes inoculated and uninoculated communities. Based on MDA, the total density of RF-based discriminant genera was highly abundant in active compost samples collected from dairy farm #1 and finished compost from poultry farm #2. Specifically, *Bacillus* and *Geobacillus* were predominant in sample collected from dairy farm #1, whereas *Lentibacillus*, *Bacillus*, and *Brevibacterium* were predominant in sample collected from poultry farm #2.

### Shotgun metagenomics reveal functional capacity of dairy compost microbiome.

For shotgun approach, the compost samples were provided by dairy farm #1 from two collections, which had the same compost-related parameters (e.g., compost ingredients, composting length) as the compost samples used for amplicon sequencing. Based on the taxonomic results from shotgun metagenomics, active dairy compost was dominated by bacteria (98.14%), followed by archaea (0.97%), eukaryote (0.53%), virus (0.01%), and other unassigned sequences (< 0.01%) (Data not shown). The five most abundant phyla in active dairy compost collected from dairy farm #1 are *Firmicutes*, *Actinobacteria*, *Proteobacteria*, *Chloroflexi*, and *Bacteroidetes,* and these five phyla account for at least 91% of all classified reads in active dairy compost samples (Fig. S3A in the supplemental material). Clearly, the five most abundant phyla in active dairy compost collected from dairy farm #1 agrees with those measured using 16S rRNA gene sequencing (Fig. 1).

Based on functional profiling, clustering-based subsystems, carbohydrate metabolism, and amino acids and derivatives have the largest quantity of annotated reads assigned in the active dairy compost samples, representing 15, 13, and 10%, respectively (Fig. S3B in the supplemental material). To gain a comprehensive understanding of the functional capacities of active dairy compost from farm #1, two separated collections from two composting rows with the same ingredients and composting length were analyzed. Based on SEED subsystem level 1, 8 of the 28 functional profiles were found to be significantly different (BH-FDR adjusted *P < *0.05) between two collections (Fig. S4). The functional profiles associated with DNA metabolism and motility and chemotaxis were found to be higher (BH-FDR adjusted *P < *0.05) in relative proportions in collection A compared to collection B, whereas fatty acids, lipids, and isoprenoids were higher (BH-FDR adjusted *P < *0.05) in relative proportions in the collection B.

### L. monocytogenes inoculation is associated with community functional changes in active dairy compost as revealed by metagenomic sequencing.

**FIG 4:**
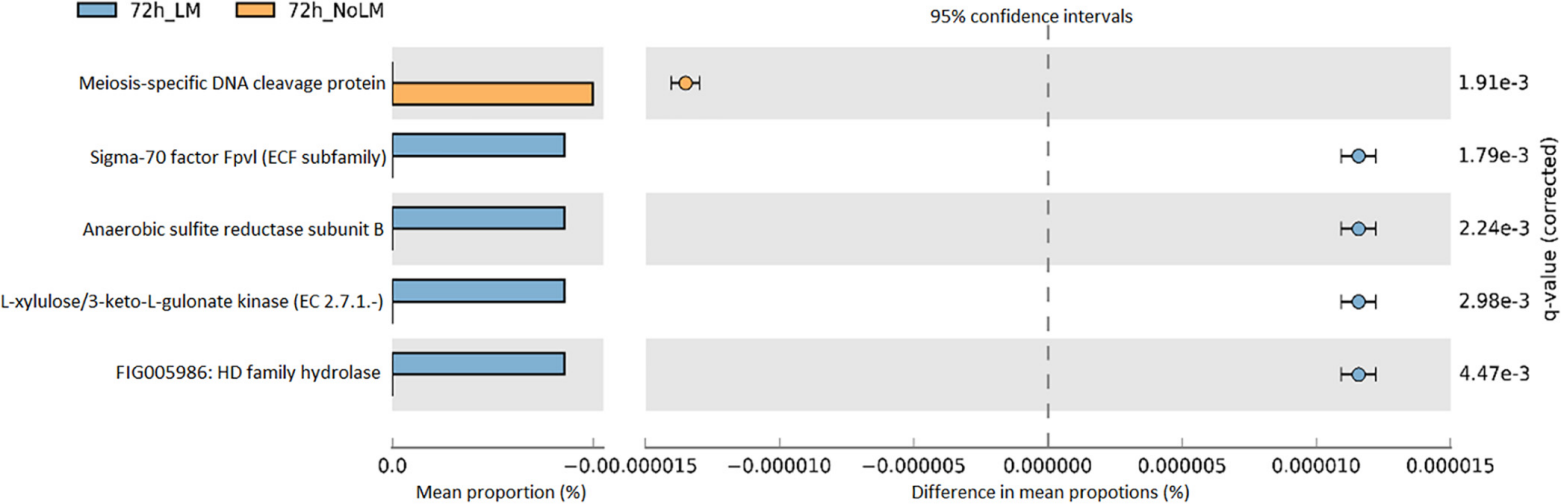
Extended error bar plot indicating the microbial functional potentials changed in active dairy compost from collection B due to the inoculation of L. monocytogenes (LM) after 72 h of incubation based on the SEED subsystem function genes entries. Points and bars indicate the differences between L. monocytogenes inoculated- and uninoculated- compost (light blue and light orange, respectively), and the values at the right show the *P*-values were derived from a White’s non-parametric *t* test with Benjamini–Hochberg FDR correction.

The microbial functional profiles of composts, with and without inoculation of L. monocytogenes as classified at SEED functional gene entries, were further analyzed separately for the two collections at 72 h of incubation. For collection B, genes that are assigned to different functional roles including pyoverdine biosynthesis, anaerobic sulfite reductase subunit, L-ascorbate utilization, and divergent RNA modification related cluster (HD family hydrolase) were significantly (BH-FDR adjusted *P < *0.05) enriched in compost samples inoculated with L. monocytogenes, whereas meiosis-specific DNA cleavage protein was found to be more abundant in proportions in the compost samples without L. monocytogenes. These observations were consistent among the technical replicates in collection B (Fig. 4). Overall, for collection B, the core functional capacities at subsystem level 2 of compost microorganisms changed due to L. monocytogenes inoculation belong to different subsystem level 1 categories, including iron acquisition and metabolism, DNA metabolism, respiration, and carbohydrate metabolism. However, the inoculation of L. monocytogenes did not induce the change in the aforementioned functional capacities of compost microbiome from collection A. These results were not surprising because the functional profiles of compost samples from these two collections were statistically different.

### L. monocytogenes inoculation is associated with distinct changes to microbial gene expression in active dairy compost as revealed by metatranscriptomic sequencing (collection B).

A multiple-group comparison was used to identify the pathways whose expression levels were not the same across all treatment groups (compost sample inoculated with or without L. monocytogenes at 0 or 72 h of incubation periods). According to the annotation with SEED level 1 Subsystems of MG-RAST, abundant categories including clustering-based subsystems, amino acids and derivatives, cell wall and capsule, DNA metabolism, and virulence, disease and defense changed significantly among different treatment groups (Table 4).

**TABLE 4:** Relative proportions of selected functional categories found in active dairy compost samples that significantly changed among different treatments annotated by SEED subsystem level 1 and level 2 as revealed by metatranscriptomic sequencing^*a*^

Functional categories		No LM			With LM	
Level 1	Level 2	0h (%)	72h (%)		0h (%)	72h (%)
Amino acids and derivatives	Arginine; urea cycle, polyamines	8.02 ± 0.02	9.65 ± 0.34		7.85 ± 0.12	**11.7 ± 0.18** ^*b*^
Cell wall and capsule	Gram-positive cell wall components	1.17 ± 0.03	2.2 ± 0.01		1.06 ± 0.17	**3.3 ± 0.11**
Clustering-based subsystems	Hypothetical related to dihydroorate dehydrogenase	Low in proportions	Low in proportions		Low in proportions	Low in proportions
Clustering-based subsystems	Two related proteases	0.73 ± 0.01	0.92 ± 0.02		0.69 ± 0.02	0.76 ± 0.01
DNA metabolism	DNA repair	62.35 ± 0.05	61.11 ± 0.4		62.98 ± 0.16	56.84 ± 0.12
DNA metabolism	Unclassified	4.87 ± 0.07	6.73 ± 0		4.79 ± 0.12	**9.07 ± 0.44**
Phages, prophages, transposable elements, plasmids	Phages, prophages	17.69 ± 2	48.57 ± 3.2		15.11 ± 0.18	**78.81 ± 1.21**
Phages, prophages, transposable elements, plasmids	Pathogenicity islands	78.97 ± 2.1	48.21 ± 3.97		82.23 ± 0.52	17.76 ± 0.6
Virulence, disease and defense	Resistance to antibiotics and toxic compounds	79.55 ± 0.94	84.09 ± 0.35		87.89 ± 0.24	**88.2 ± 0.13**

a The numbers highlighted in bold indicate a significant (*P*-value < 0.001) increase in the presence of L. monocytogenes after 72 h incubation.

b The bold numbers are indicating the significant levels (BH-FDR adjusted *P*-value) which are less than 0.05.

Further, 75 of 196 pathways of active functional categories were found at the SEED subsystem level 2 to be differentially expressed from multiple-group comparison. Next, we used a *post hoc* test to identify which pairs of groups differ from each. The categories that significantly increased due to the inoculation of L. monocytogenes after 72 h of incubation (BH-FDR adjusted *P < *0.001) are presented in Table 4. In contrast, at 0 h postinoculation, the mean proportions of the above pathways in compost samples with or without L. monocytogenes did not differ significantly (*P ≥ *0.05).

**FIG 5:**
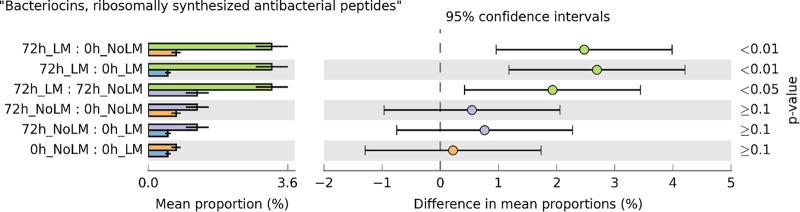
Post-hoc plot for bacteriocins, ribosomally synthesized antibacterial peptides, as revealed by metatranscriptomic sequencing. The light blue, orange, green, and purple bars indicate compost samples at 0 h with L. monocytogenes (LM), 0 h without LM, 72 h with LM, and 72 h without LM, respectively.

Knowing that the significant changes in pathways were occurred after 72 h of incubation, 40-three active function roles that significantly associated with the inoculation of L. monocytogenes after 72 h were shown in Fig. S5 in the supplemental material [BH-FDR corrected *P*-value < 0.05, ROTS test with rare entries (*n* < 5) were removed from the analysis]. SEED-annotated functions were also clustered into 23 SEED subsystems level 2 and 17 SEED subsystem level 1, based on SEED hierarchical clustering. Functions such as bacteriocins, ribosomally synthesized antibacterial peptides, and resistance to antibiotics and toxic compounds were relatively higher in the compost sample inoculated with L. monocytogenes compared to the sample without L. monocytogenes. Both NADH dehydrogenase subunit 4 (EC:1.6.99.3) and negative regulator of genetic competence were the most active functional roles in compost samples with L. monocytogenes. Specifically, the mean proportions of microbial functions associated with bacteriocins, ribosomally synthesized antibacterial peptides, significantly (*P < *0.05) increased in the compost sample with L. monocytogenes after 72 h of incubation, with a higher mean proportion (Fig. 5). The actively expressed genes related to ABC transporter ATP-binding protein (bacteriocins, ribosomally synthesized antibacterial peptides), arsenic efflux pump protein (resistance to antibiotics and toxic compounds), and negative regulator of genetic competence (DNA uptake, competence) were mapped to *Bacillus* spp., Staphylococcus haemolyticus, and *Geobacillus* spp., respectively. And the NADH dehydrogenase subunit 4 function was mapped into multiple species of bacteria.

## DISCUSSION

Overall, the most abundant phyla and orders of compost microbiomes agreed with those reported in previous studies (20–22). In active dairy and poultry composts, the primary indicator genera were *Salinicoccus* and *Ureibacillu*s, respectively, which belong to *Firmicutes*. As most species in *Firmicutes* are more adapted to the thermophilic conditions (23, 24), there was higher relative abundance of *Firmicutes* observed in active composts compared to those in finished composts in most compost samples collected in this study. However, *Firmicutes* was the most abundant phylum in the finished compost collected from poultry farm #2 as well. This inconsistent observation in poultry farm #2 was likely due to the fact that the temperature of the finished compost pile on that farm was still very high (53.3°C) compared to the temperature of the finished compost samples collected from other farms, suggesting the active composting process indeed.

In the finished compost, *Chloroflexi* represents a significantly greater proportion of sequence reads in finished dairy compost compared with active dairy, active and finished poultry compost. This observation is consistent with other studies reporting the increased abundance of *Chloroflexi* during the maturation phase of composting (13, 24). The increase in abundance of *Chloroflexi* and *Proteobacteria* in the finished dairy compost may also be attributed to their ability to decompose lignocellulose, cellulose, lignin, and other complex organic compounds in dairy manure (22, 25). Furthermore, the indicator genera in finished poultry compost, including *Chryseolinea*, *Hyphomicrobium*, and *Planctomyces*, were identified previously in composted chicken manure (26, 27). These genera are known to be involved in nitrogen cycling or positively correlated with the presence of nitrate (NO_3_^−^). Additionally, it is not surprising that the microbial composition in compost was unique to each compost farm (Fig. 3). Given the fact that the compost-related factors are the major drivers that affected the microbial compositions in compost, it is highly likely that the variance in microbial communities and function profiles of animal waste-based compost was due to the heterogeneous nature of compost ingredients at the start of composting process.

To understand the changes in artificially inoculated L. monocytogenes populations in different compost samples, a relatively high-level of L. monocytogenes (ca. 7 log CFU/g) was inoculated into the compost samples. For the finished compost sample collected from poultry farm #2, the pathogen regrowth under higher moisture level (80%) might be due to the much lower populations in total bacterial counts, heterotrophs, thermophiles, and actinomyces (Table S2 in the supplemental material). And the low counts of heterotrophs and actinomyces were also observed in compost samples collected from dairy farm #1. In addition to the culturable microorganisms, the interactions from the microbiome with invading pathogens could come from those uncultured compost microorganisms, which account for >90% of total microorganisms in many ecosystems (28). Studies have been conducted to elucidate that the role of indigenous compost microorganisms in suppression of human pathogens including L. monocytogenes, Salmonella, and E. coli O157: H7 (29–31). Kim and Jiang (31) reported that the regrowth of E. coli O157: H7, Salmonella spp., and L. monocytogenes occurred in autoclaved dairy compost with 2.1- to 3.9-log increases, but not in dairy manure compost (40% moisture content) with 6.5 log CFU/g of indigenous microbiota. Some microbial species in indigenous population may play antagonistic role against the invading pathogens. Considering the complexity of composting process, other factors, including other microorganisms and the manure types, could have impacted the fate of pathogens in compost. For example, Neher et al. (32) found that E. coli only persisted in compost amended with poultry litter but not in the compost containing dairy manure.

Random Forest-based discriminant analysis showed that important genera such as *Bacillus*, *Geobacillus*, *Lentibacillus*, and *Brevibacterium,* can be the biomarkers that classify the compost samples into *Listeria*-inoculated and uninoculated samples. However, the changes in the compost microbiome composition induced by the inoculation of L. monocytogenes were too subtle and inconsistent across farms, resulting in a lack of strong statistical significance. This is likely due to the robustness and the resilience of the resident microbiome of the composts to perturbation. In support of this statement, Falardeau et al. (33) found that native bacterial communities in soil were not driven by the inoculation of L. monocytogenes but by differences in pH and moisture contents of natural soil samples. In other studies, effects from inoculated Salmonella (ca. 7 log CFU/g) and E. coli O157: H7 (ca. 8 log CFU/g) on the composition of the prokaryotic communities were not visible in the untreated-sandy, clay, or regular soil samples (34, 35). Xing et al. (34) found that phylogenetic diversity decreased by 43.6% due to the inoculated E. coli O157:H7 in irradiated soils. In another study performed by Schierstaedt et al. (35), the effect of Salmonella on the microbial community was observed only in the autoclaved soils. However, findings about the microbial interactions with invading pathogens from the afore-mentioned studies were simplified by manipulating an environmental microcosm (e.g., irrigation, dilution to extinct, or autoclaving), leading to the severe reduction on the microbial diversity.

Additionally, even the changes in the composition of the indigenous compost microbiome due to L. monocytogenes were very subtle, there were significant changes in the functional composition and gene expression. As revealed by metatranscriptomic sequencing, the most strongly enriched gene category in the compost samples inoculated with L. monocytogenes was NADH dehydrogenase subunit 4 (EC:1.6.99.3), which is a critical regulator to maintain homeostasis in most bacteria (36). Although the expression of NADH dehydrogenase was not mapped specific to L. monocytogenes, we hypothesized that the change in expression for this gene may be responsible for the pathogen intrusion, as the expression of NADH dehydrogenase is one of the important redox-responsive regulators in L. monocytogenes or other Gram-positive bacteria to survive in the stressed environment (36). And negative regulator of genetic competence was another strongly expressed gene in compost sample spiked with L. monocytogenes. Based on the mapping results, the negative regulator of genetic competence was associated with *Geobacillus* spp., which suggests a potential competitive activity from *Geobacillus* spp. against L. monocytogenes. Consistent with this observation, Ottesen et al. (37) reported that *Geobacillus* spp. outcompeted with L. monocytogenes in ice cream during 4–12 h of enrichment. Moreover, in agreement with the results from 16S rRNA genes sequencing, the increased gene abundance in bacteriocins upon the introduction of L. monocytogenes suggests that *Bacillus* spp. or other species from active compost might have antagonistic activity against the invading pathogen by producing antimicrobial peptides.

This is the first study to understand the compositional and functional changes in compost microbiome as affected by the intrusion of L. monocytogenes using metagenomics approaches. However, there are some limitations in our study. Due to the cost of shotgun sequencing analysis, we were unable to process all samples for shotgun approach. Therefore, we used the two-step approach in which a large number of samples was analyzed using 16S rRNA gene sequencing first, and then a subset of samples was further analyzed by the shotgun approaches. Due to the concern of RNA degradation during storage, compost samples (dairy farm #1) were freshly collected for shotgun sequencing separately. Besides, the extracted RNA was pooled from multiple extractions to reach the required concentration. We suspect that using only a single dairy farm for compost sample analysis may have contributed to detecting more consistent differences. Therefore, the results from metatranscriptomic sequencing should be interpreted with caution due to the insufficient biological replicates. Although the diversity of taxa that classify compost sample into L. monocytogenes inoculated and uninoculated samples was identified, a further study is needed to use culture methods to isolate the CE microorganisms with anti-listeria activities from compost.

The microbial diversity, structure and functions varied among compost samples and were significantly affected by the composting-related factors. Our study has provided a comprehensive analysis of compost microbiome at both compositional and functional levels using high-throughput sequencing approaches. Further analysis using Random Forest has identified some discriminatory species in compost inoculated with and without L. monocytogenes. Findings from this study can be useful for the composting industry to understand the composition and functionality of microbial community in their products better, and to interrogate those discriminatory microbial members from compost samples as some of them could be leveraged as natural deterrents in the organic fertilizer.

## MATERIALS AND METHODS

### Compost sample collection.

A total of 12 biological soil amendments (6 dairy- and 6 poultry waste-based composts) were collected from 6 different facilities. Those facilities are in Arizona, California, Michigan, South Carolina (*n* = 2), and Wisconsin, United States. Each facility provided composting samples at two stages within the composting process: thermophilic composting stage (active compost, >55°C/131°F, within 1 month of composting) and finished stage (finished compost, 3 to 6 months of composting). Major compost ingredients included animal manure, sawdust, cow paunch, green waste, and agricultural by-products. Following the sampling protocol recommended by the California Leafy Greens Marketing Agreement (38), samples were collected in Ziploc bags, shipped under the ambient condition to our lab, and then stored at refrigeration conditions (4°C) once received. Importantly, as a precaution against potential freeze-damage to cell membranes, compost samples were deliberately not frozen. To reduce the DNA degradation and microbial population change, the sample preparation and microbiological analysis for most samples were performed within 7 days after the samples were received.

### Microbiological and physicochemical analyses of compost samples.

Compost samples were analyzed for total aerobic bacteria, actinomyces, yeast/mold, *Enterobacteriaceae*, thermophilic, and heterotrophic bacteria by plating serial dilutions onto 3M Petrifilm aerobic count plates (3M, USA), Actinomycete Isolation Agar (AIA; Becton, Dickinson and Company; NJ, USA), Rose Bengal Agar (RBA; Hardy Diagnostic; CA), Violet Red Bile Glucose Agar (VRBG; Hardy Diagnostic), tryptic soy agar (TSA; BD) and Reasoner's 2A agar (R2A; BD), respectively, followed by incubation at 35°C for 24 h, 25°C for 48 h, 25°C for 5 days, 35°C for 24 h, 55°C for 24 h, and 25°C for 5–7 days, respectively. Samples were also examined for the presence of background L. monocytogenes by following Food and Drug Administration’s Bacteriological Analytical Manual (39) procedure and enumerated onto Oxford agar (Hardy Diagnostic). Briefly, the samples were examined by directly plating and enrichment procedure. The limit of detection of the procedure is 1 CFU per 25 g of compost sample. DNA was extracted from the black colonies grown on Oxford agar (Difco, BD, Sparks, MD) and analyzed by PCR assay that targeted the *hly*A gene (40).

Moisture contents of compost samples were measured with a moisture analyzer (model IR-35, Denver Instrument, Denver, CO), whereas pH values were measured based on the methods described by U.S. Composting Council (40, 41). Additionally, compost samples in duplicate were analyzed by Clemson Agricultural Service Laboratory for chemical characterization, including total nitrogen, carbon, organic matter, and soluble salts.

### Compost inoculation and L. monocytogenes enumeration.

The experimental designs for compost inoculation and sequencing analysis are shown in Fig. S6 in the supplemental material. Three portions (ca. 200 g per portion) from each compost sample served as technical replicates for the subsequent experiments. The compost samples were thoroughly mixed and adjusted to 40 or 80% moisture contents with autoclaved tap water and half of the samples were artificially inoculated with L. monocytogenes strain FSL R9-5506 (a pathogenic strain isolated from packaged salad, kindly provided by Dr. Martin Wiedmann at Cornell University). To prepare for the inoculum, the L. monocytogenes culture was streaked twice onto TSA, and then grown overnight in tryptic soy broth (TSB) at 35°C, followed by washing and resuspending in 0.85% saline to ca. 10^9^ CFU/mL. Afterwards, the culture was inoculated into the compost samples with the target moisture contents (40 or 80%), mixed well by hand at a final inoculation level of ca. 7 log CFU/g, and then held in a sterile aluminum tray (ca. 22 × 16×16 cm) covered loosely by sterile aluminum foil, followed by incubation at room temperature. At 0 and 72 h postinoculation, L. monocytogenes population in each compost sample was enumerated by plating 10-fold serial dilutions onto Oxford media plates, followed by incubation at 35°C for 24 h.

### DNA and RNA extraction.

Compost samples from multiple farms around the US were collected and analyzed to identify the most common microbes associated with compost products. DNA was extracted from all compost samples and used for 16S rRNA gene sequencing (Fig. S6). For shotgun approach, dairy farm #1 was able to provide the compost samples from two separated collections from two composting rows, which had the same compost-related parameters (e.g., compost ingredients, composting length) to the compost samples used for amplicon sequencing. Therefore, the compost samples were freshly requested from dairy farm #1 for the subsequent shotgun-metagenomics and metatranscriptomic sequencing. Both DNA and RNA were extracted from fresh active compost samples from dairy farm #1. The total DNA and RNA were extracted using the ZymoBIOMICS DNA and DNA/RNA miniprep extraction kits (Zymo Research, Irvine, CA, USA), respectively, according to manufacturer’s instructions. *DNase I* treatment step was included during RNA extraction for DNA removal.

For PMA treatment (18, 42), the compost slurry (1:4 wt/vol) was transferred to a transparent 2-mL microcentrifuge tube and mixed with propidium monoazide (PMA dye, Biotium, Inc. CA, USA) at a final concentration of 50 μM in a dark room and incubated for 5 min on a rotating mixer at room temperature. Next, the microcentrifuge tube was subsequently placed on ice horizontally and exposed to 650 W halogen light source at 20 cm distance for 20 min. Afterwards, PMA was removed by centrifugation at 12,000 × *g* for 5 min and DNA was extracted immediately from the pellet.

RNA was further purified using the RNA Clean & Concentrator kit (Zymo Research, Irvine, CA). The purity and concentration of both DNA and RNA were evaluated on a NanoDrop -2000 spectrophotometer (NanoDrop Technologies, DE, USA) at 260, 280, and 230 nm. Further quantification of DNA and RNA was performed using Qubit fluorometer (Thermo Fisher, MA, USA). RNA integrity number (RIN) was determined using an RNA 6000 Nano kit in the 2100 Bioanalyzer (Agilent Technologies, USA). Upon completion of extraction, samples were submitted to the respective sequencing facilities for 16S rRNA gene, shotgun-metagenomic, or metatranscriptomic sequencing analysis as described below.

### 16S rRNA gene sequencing and sequencing data processing.

In total, 288 gDNA samples were submitted to ZymoBIOMICS Services (Zymo Research, Irvine, CA) for library preparation and bacterial 16S rRNA gene sequencing. Bacterial 16S rRNA gene targeted sequencing was prepared using the Quick-16S NGS library prep kit. The bacterial 16S primers were used to amplify the V3-V4 region of the 16S rRNA gene. The final pooled library was cleaned up with the Select-a-Size DNA Clean & Concentrator (Zymo Research, Irvine, CA), then quantified with TapeStation (Agilent Technologies, Santa Clara, CA) and Qubit. The final library was sequenced on Illumina MiSeq with a Version 3 reagent kit (600 cycles). The sequencing was performed with >10% PhiX spike-in.

Bioinformatics analysis was performed as following according to Zymo research: Unique amplicon sequences were inferred from raw reads and chimeric sequences were removed using the DADA2 pipeline (43). Taxonomy annotation was performed using Qiime V.1.9.1 with the Zymo Research database (43, 44), a 16S database that is internally designed and curated, as reference. We used even-sampling of raw counts to adjust for unequal sequencing depth. Finally, each taxonomy was adjusted for 16S copy number variation in order to represent true relative abundances (45). The 16S copy number adjustment was performed using copy number estimates from the rRNA Database (rrnDB) v5.4.

### Shotgun-metagenomics and metatranscriptomic sequencing.

As shown in Fig. S6, 24 DNA and 8 RNA samples were submitted to Novogene Inc. (Sacramento, CA) for shotgun-metagenomic and metatranscriptomic sequencing, respectively. For shotgun-metagenomic sequencing, sequencing libraries were generated using NEBNext DNA Library Prep Kit (New England Biolabs, Ipswich, MA) following the manufacturer's recommendations, and indices were added to each sample. For the metatranscriptomic sequencing, after the initial QC procedure, mRNA from eukaryotic organisms was enriched using oligo (dT) beads. Simultaneously, rRNA was removed using the Ribo-Zero kit (Illumina, San Diego, CA) and the bacterial mRNA was concentrated. Following the QC steps, the qualified libraries from DNA and RNA were fed into Illumina sequencers (PE 150) after pooling according to its effective concentration and expected data volume, respectively.

Reads containing adapter and low-quality base (Q-score ≤ 5) were removed from the raw reads. All the shotgun-metagenomic and metatranscriptomic sequencing data had a >99% “good reads” after filtering and trimming with AfterQC (46). Afterwards, all reads were uploaded into the MG-RAST analysis server (47) (https://www.mg-rast.org/). Paired reads were combined and subjected to quality filtering, and host sequences were depleted. The default parameters of the MG-RAST were used for the taxonomic and functional assignation of the sequences. All the Illumina reads that were shorter than 75 bases or had a median quality score below 20 were removed. The functional annotation was based on the SEED hierarchical system and Kyoto Encyclopedia of Genes and Genomes (KEGG) database.

### Statistical analyses.

Plate count data were converted to log CFU/g in dry weight. The Shapiro-Wilk test of normality was run to test for normality statistically prior to sequencing data analysis. The non-parametric analysis methods were used for the non-normal distribution data set.

For the 16S rRNA gene sequencing data analysis, within-community diversity (alpha diversity) was calculated by Chao richness and Shannon index of species, followed by Kruskal-Wallis and Wilcoxon signed-rank tests. Beta (β)-diversity, variation of microbial communities between environmental samples, was measured with ecological phylogenetic Unifrac distances (48). Prior to the analysis, the relative abundance data set at the genus level was subjected to chord transformation to account for many zero values (49). Linear discriminant analysis (LDA) effect size (LEfSe) was applied to search for biomarkers between different compost types (50). Principle-coordinate analysis (PCoA) was performed to determine whether samples associated with the same groups (compost type or composting stage, experimental moisture, incubation time, presence or absence of L. monocytogenes) clustered close to one another in multivariate space. Permutational multivariate analysis of variance (PERMANOVA) was used to test the statistical significance of group separation in PCoA with Benjamini–Hochberg False Discovery Rate adjusted *P* value (BH-FDR). Canonical correspondence analysis (CCA) was used to explicitly test whether the different experimental factors explained a significant fraction of the variation within the distance matrix. Lastly, the Random Forest (RF) (ntree = 500, mtry = 14, importance = TRUE, proximity = TRUE) was used to find key taxa contributing to the variation in community composition due to the presence of L. monocytogenes. These analyses were run in R with packages including vegan (version 2.5–7), randomforest (version 4.6–14), and phyloseq (BiocManager 1.20.16) (51–53).

For shotgun-metagenomic and metatranscriptomic sequencing data analysis, statistical comparisons of the proportions of functions among treatment groups of samples were conducted using STAMP (Statistical Analysis of Shotgun-metagenomic Profiles) software (54). Briefly, an ANOVA test was used to compare among multiple groups, followed by Tukey–Kramer *post hoc* tests. White’s non-parametric *t* test with BH-FDR correction for multiple tests were used for comparing two groups of data, and Welch’s inverted method was used to calculate 95% confidence intervals. ROTS was used to provide a rank of the functional gene expression based on their differential change due to the presence of L. monocytogenes after 72 h of incubation. Then, the expression of functional genes with significant fold changes (BH-FDR *P*-value < 0.05) in each sample was visualized by a heatmap (55).

### Availability of data and materials.

The raw 16S rRNA sequence data generated in the current study is archived in NCBI under BioProject PRJNA760887. All metagenomic and metatranscriptomic data files were uploaded to MG-RAST (https://www.mg-rast.org/linkin.cgi?project=mgp94064). All other data generated and analyzed for this study are included as supplemental material files.
