# Supplementary material for: Compositional and Functional Changes in Microbial Communities of Composts Due to the Composting-Related Factors and the Presence of Listeria monocytogenes
Source: Microbiol Spectr. 2022 Jun 15;10(4):e01845-21. doi: 10.1128/spectrum.01845-21 (PMC9430276; doi:10.1128/spectrum.01845-21)
Supplement: SUPPLEMENTAL FILE 1 — Supplemental material. Download spectrum.01845-21-s0001.pdf, PDF file, 1.3 MB [file spectrum.01845-21-s0001.pdf]

Supplemental Materials

Figure S1 Bacterial community composition in dairy (A, B) and poultry (C, D) compost at phylum level, with X-axis represents different samples, and Y- axis stands for relative percentage of each bacterial phylum. Left 12 lanes for active compost samples, and right 12 lanes for the finished compost samples with different experimental treatments, respectively, in terms of *L. monocytogenes* inoculation (N for not inoculation, Y for with inoculation), moisture contents (40 and 80%), and incubation period of 0 h (A, C) and 72 h (B, D). Each bar is an average between the three technical replicates.

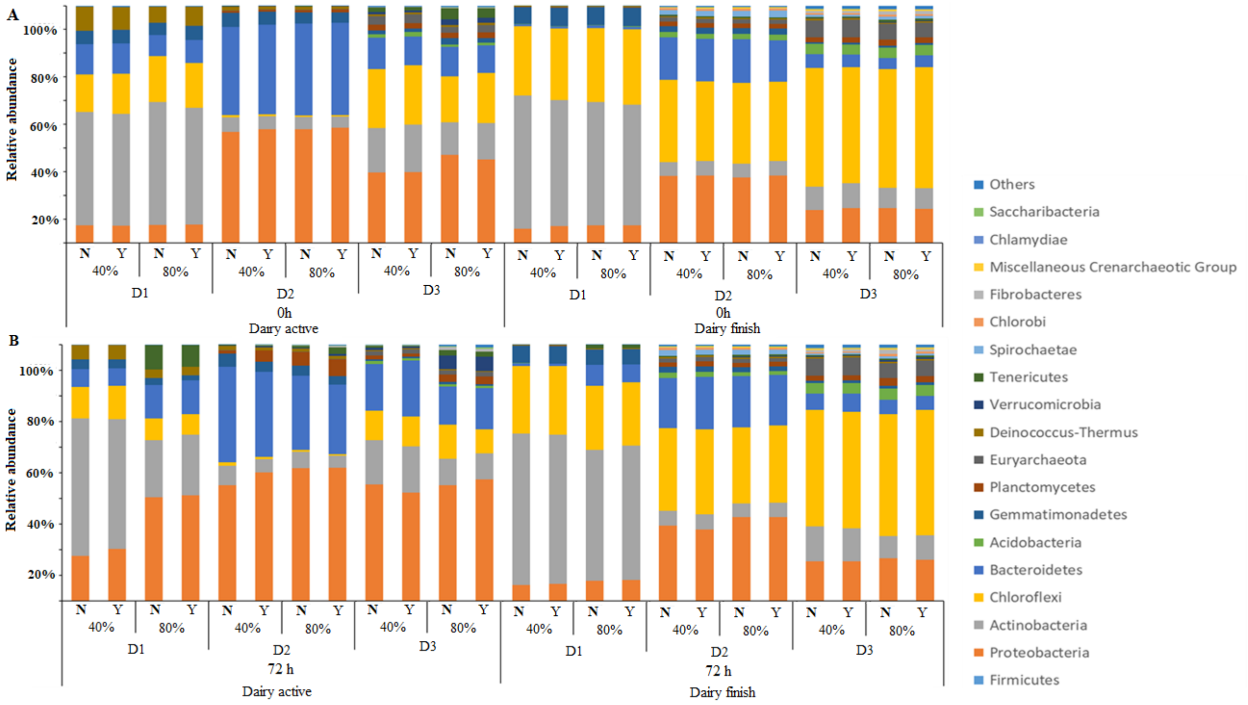

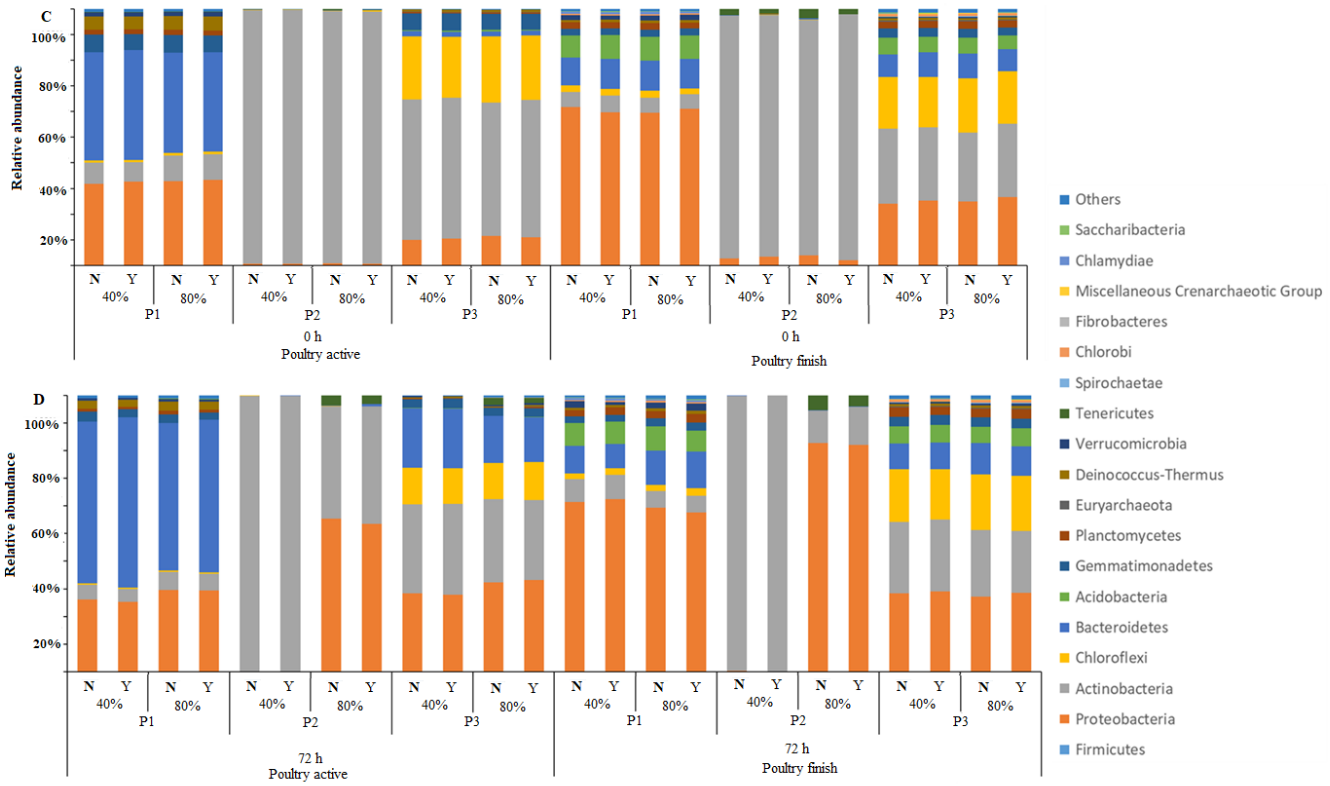
C
D
Relative abundance
100%
80%
60%
40%
20%
N
Y
40%
80%
P1
P2
P3
0 h
72 h
Poultry active
Poultry finish
Others
Saccharibacteria
Chlamydiae
Miscellaneous Crenarchaeotic Group
Fibrobacteres
Chlorobi
Spirochaetae
Tenericutes
Verrucomicrobia
Deinococcus-Thermus
Euryarchaeota
Planctomycetes
Gemmatimonadetes
Acidobacteria
Bacteroidetes
Chloroflexi
Actinobacteria
Proteobacteria
Firmicutes

Figure S2 Random Forest (RF) analysis screening top 20 variable importance in dairy and poultry compost samples collected from six farms (A-F) that driving variation in community composition due to the presence of *L. monocytogenes* after 72 h incubation periods. Mean Decrease Accuracy and Mean Decrease Gini of attributes were assigned by the RF analysis.

A RF_dairy farm 1

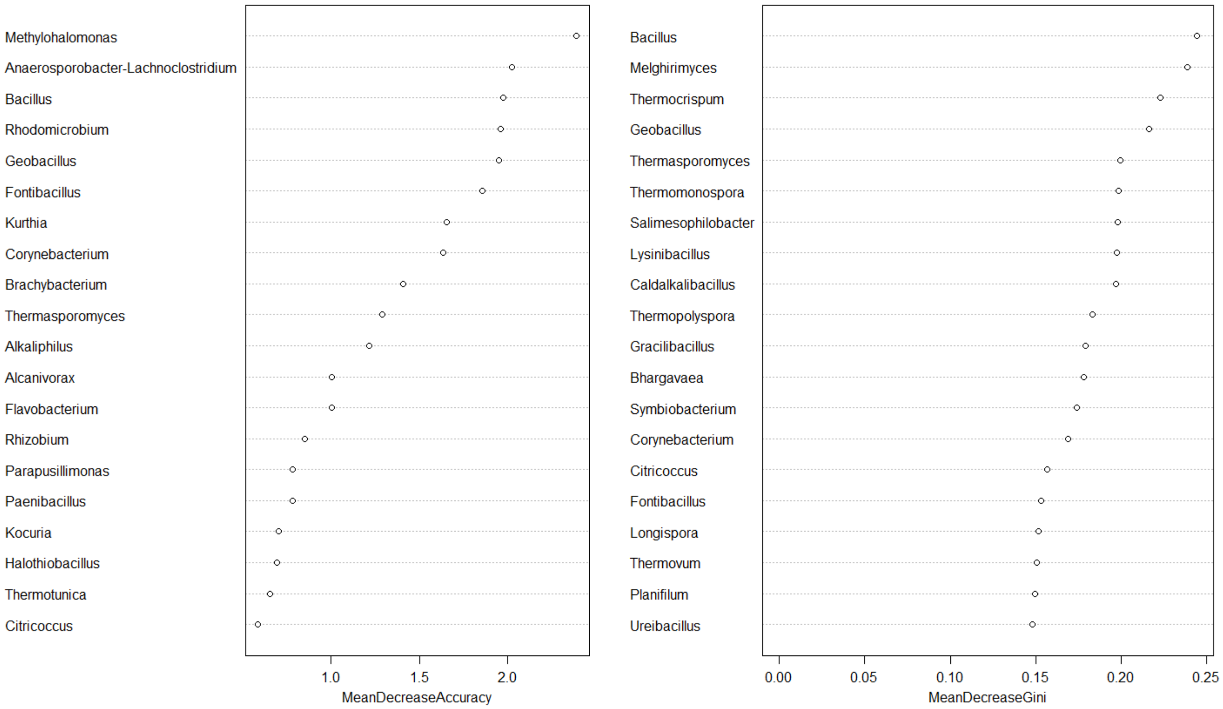

B RF_dairy farm 2

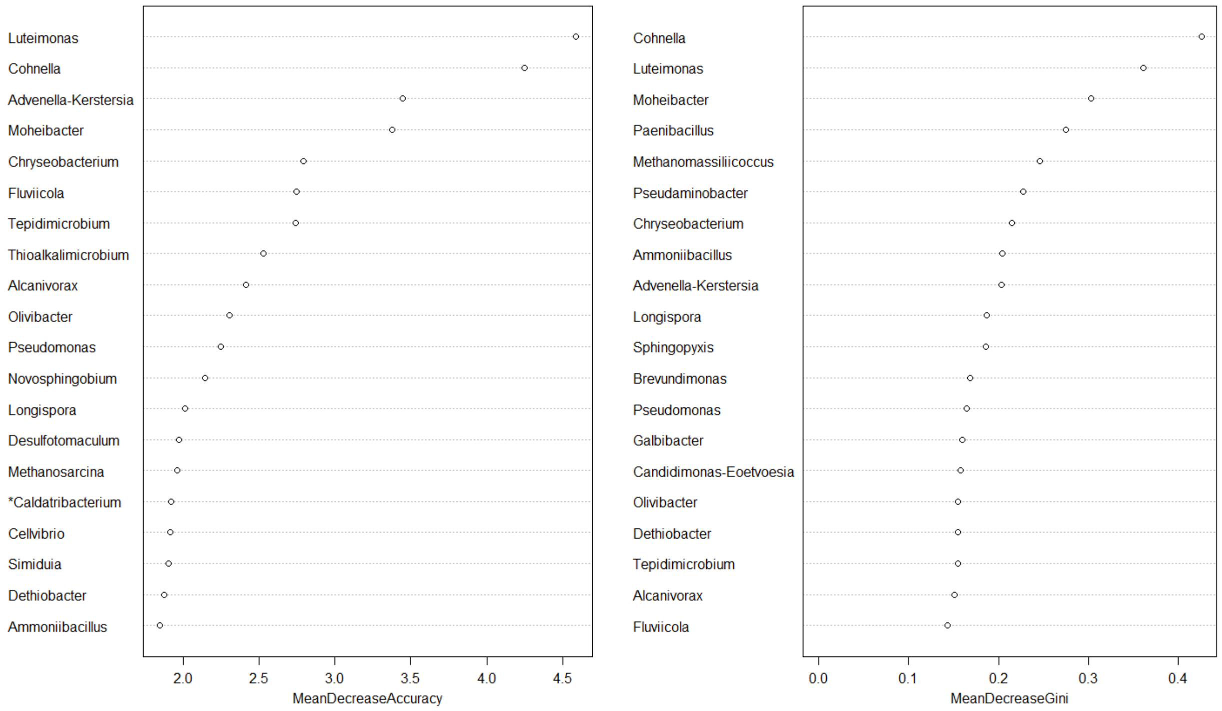

C RF_dairy farm 3

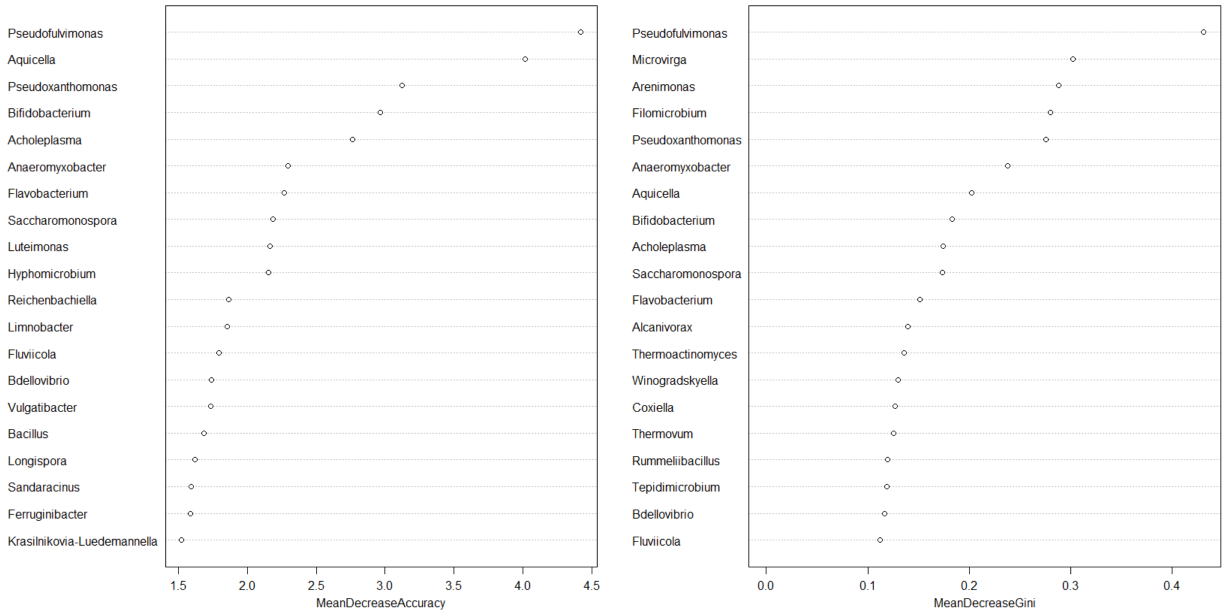

D RF_poultry farm 1

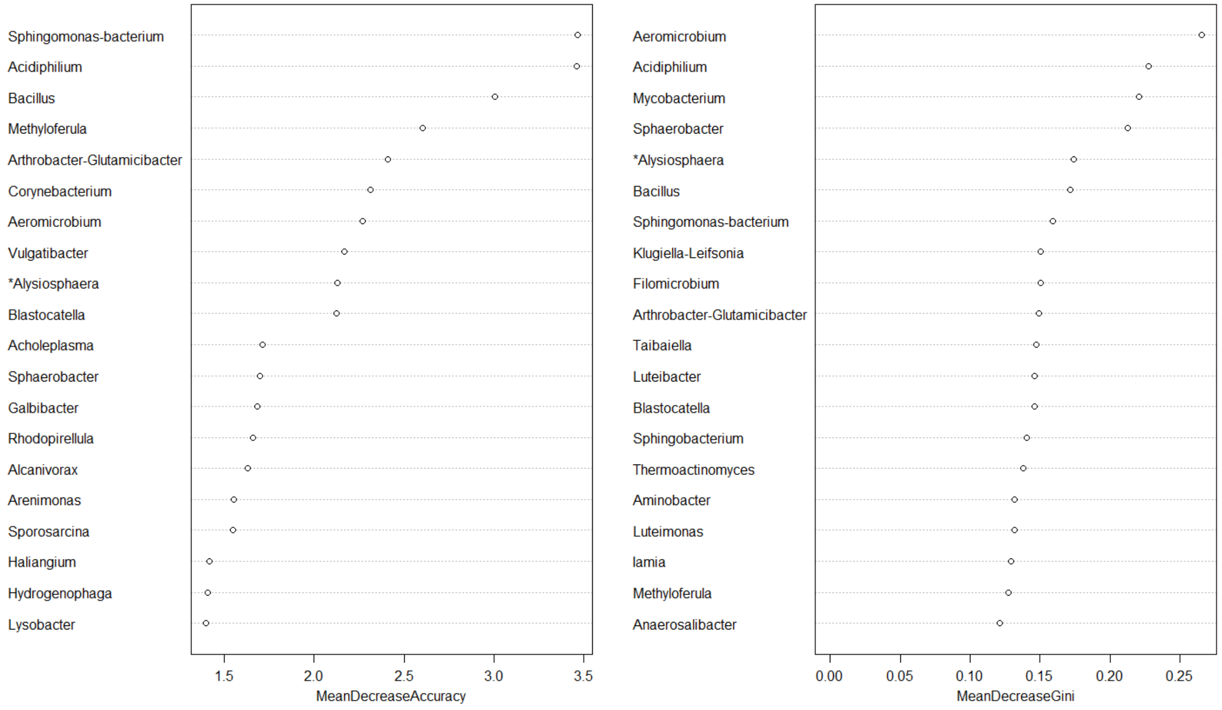

E RF_ poultry farm 2

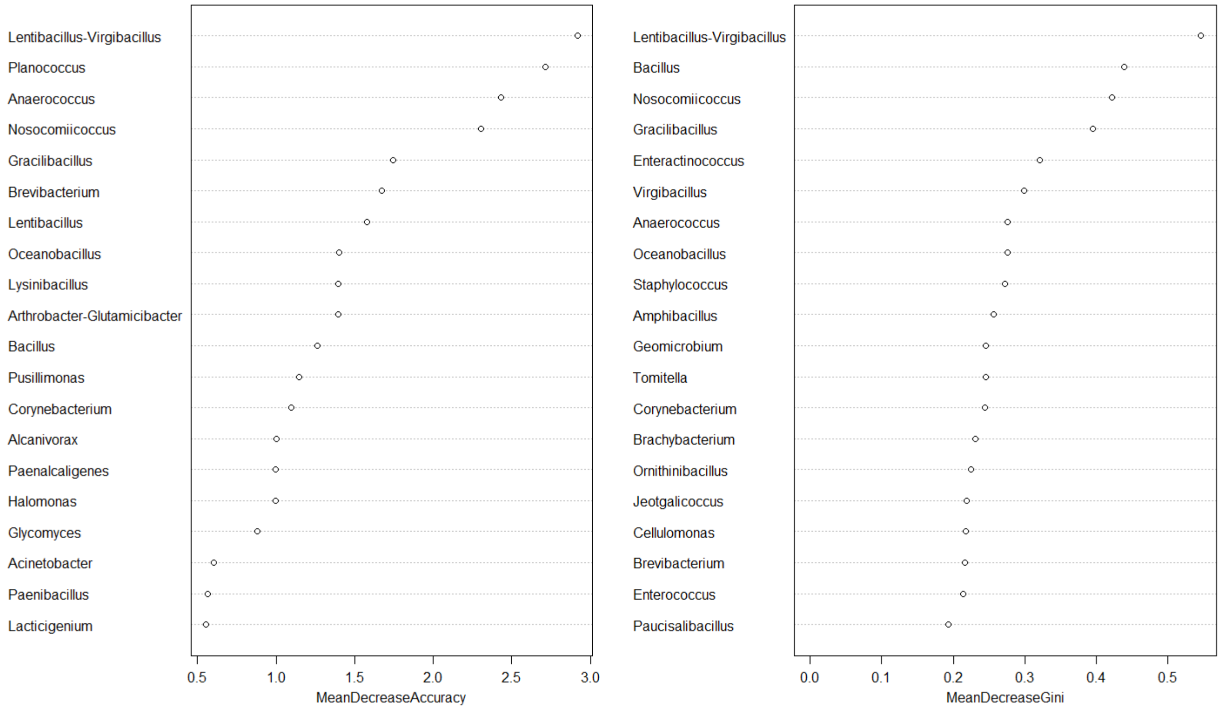

F RF_ poultry farm 3

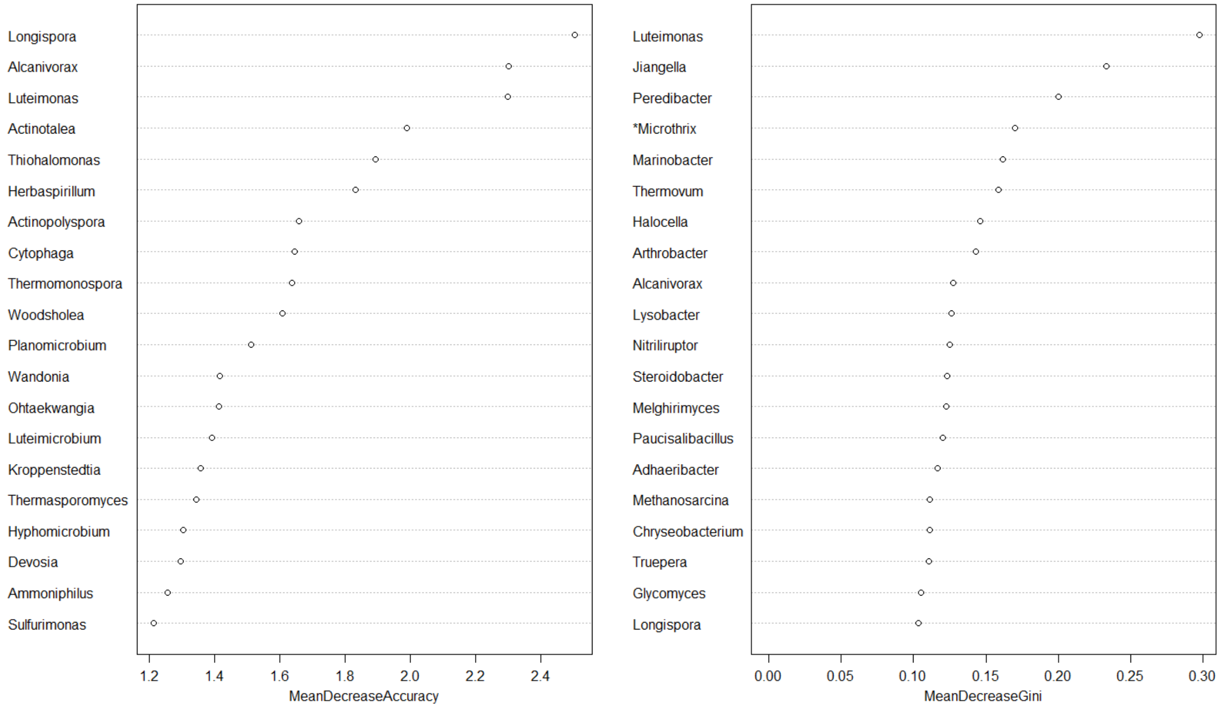

Figure S3 The five most abundant phyla in active dairy compost collected from dairy farm #1 (A) and functional profiles detected in active dairy compost microbiome annotated with SEED subsystem (level 1) (B).

A

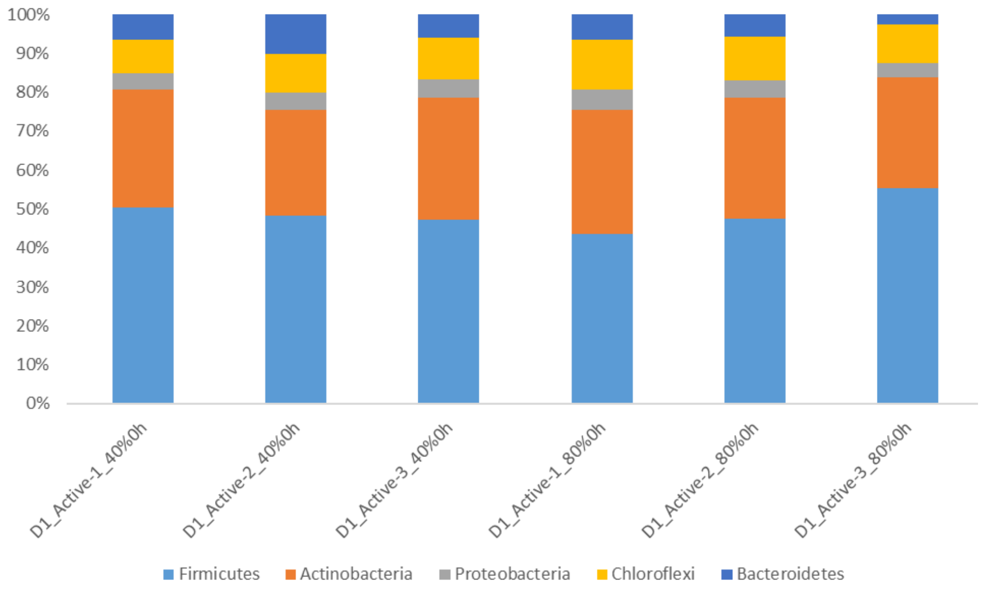

B

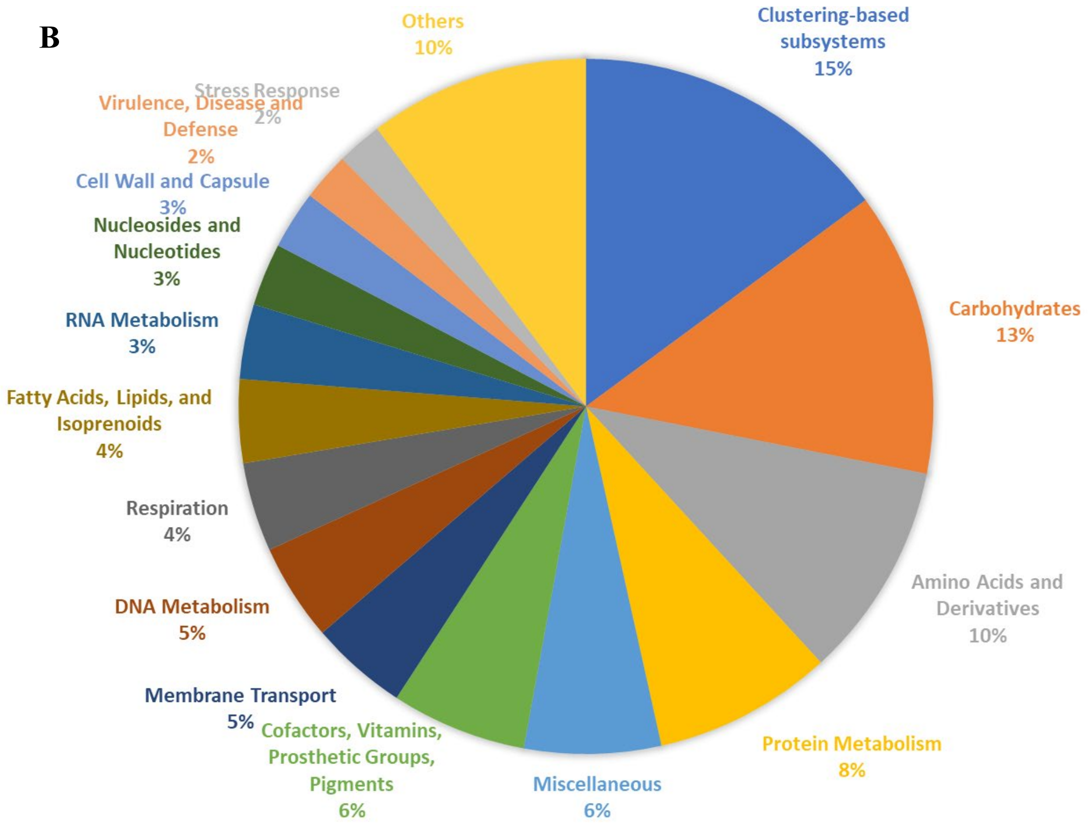

Figure S4 Functional profiles for the microbial metagenomes of active dairy compost from two separate collections (A and B). Extended error bar plot compared the functional profiles for the microbial metagenomes in active dairy compost from two separate collections based on the SEED subsystem level 1. Points and bars indicate the differences between collections A and B (blue and red, respectively), and the values at the right show the *P*-values were derived from a White's non-parametric t-test with Benjamini–Hochberg FDR correction.

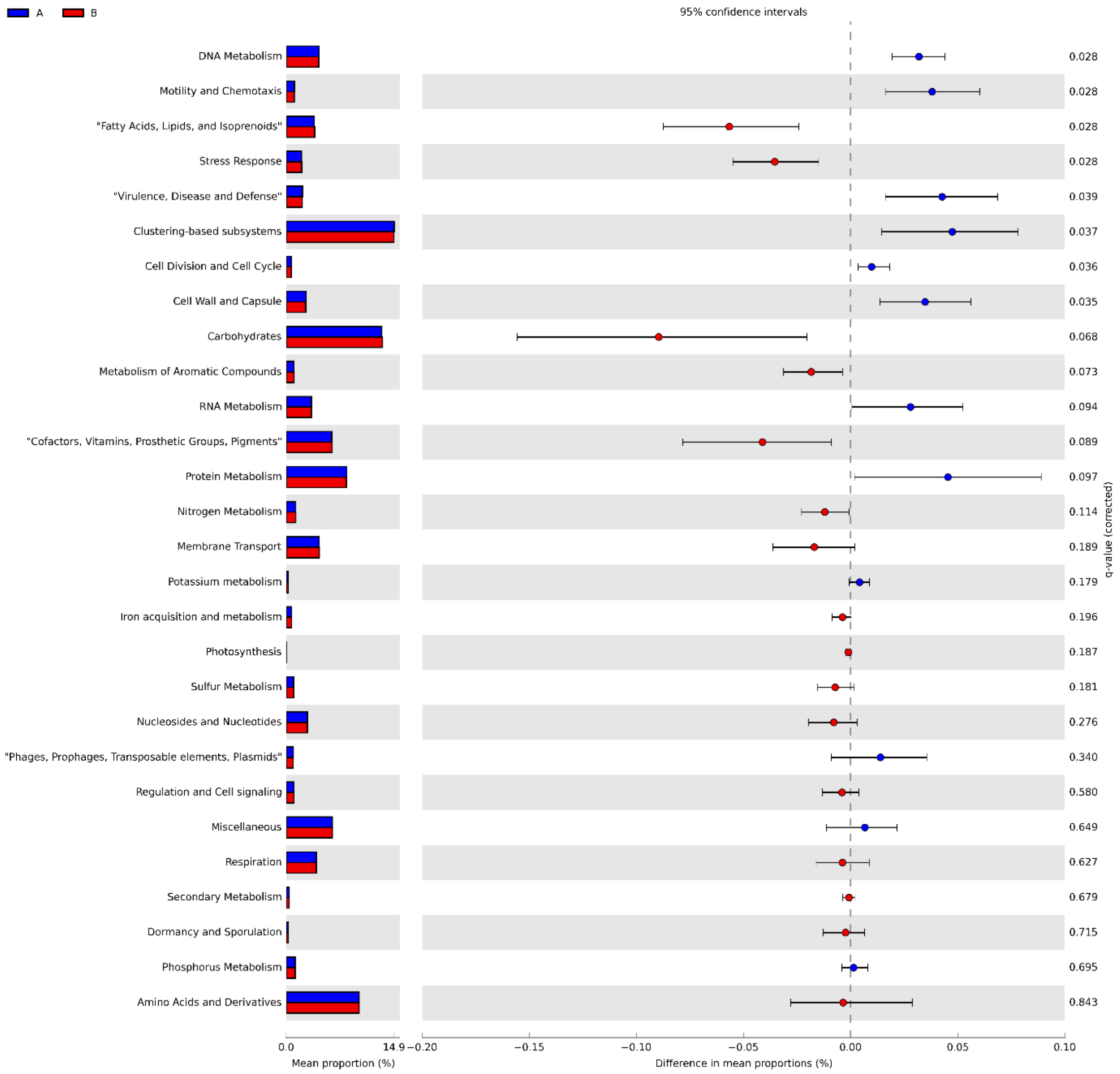

Figure S5 Genetic profile of the 43 significantly differentially abundant functional roles between *L. monocytogenes* inoculated and uninoculated group as shown in heatmap, the function list was clustered to hierarchy SEED subsystem level 2 as well (left column).

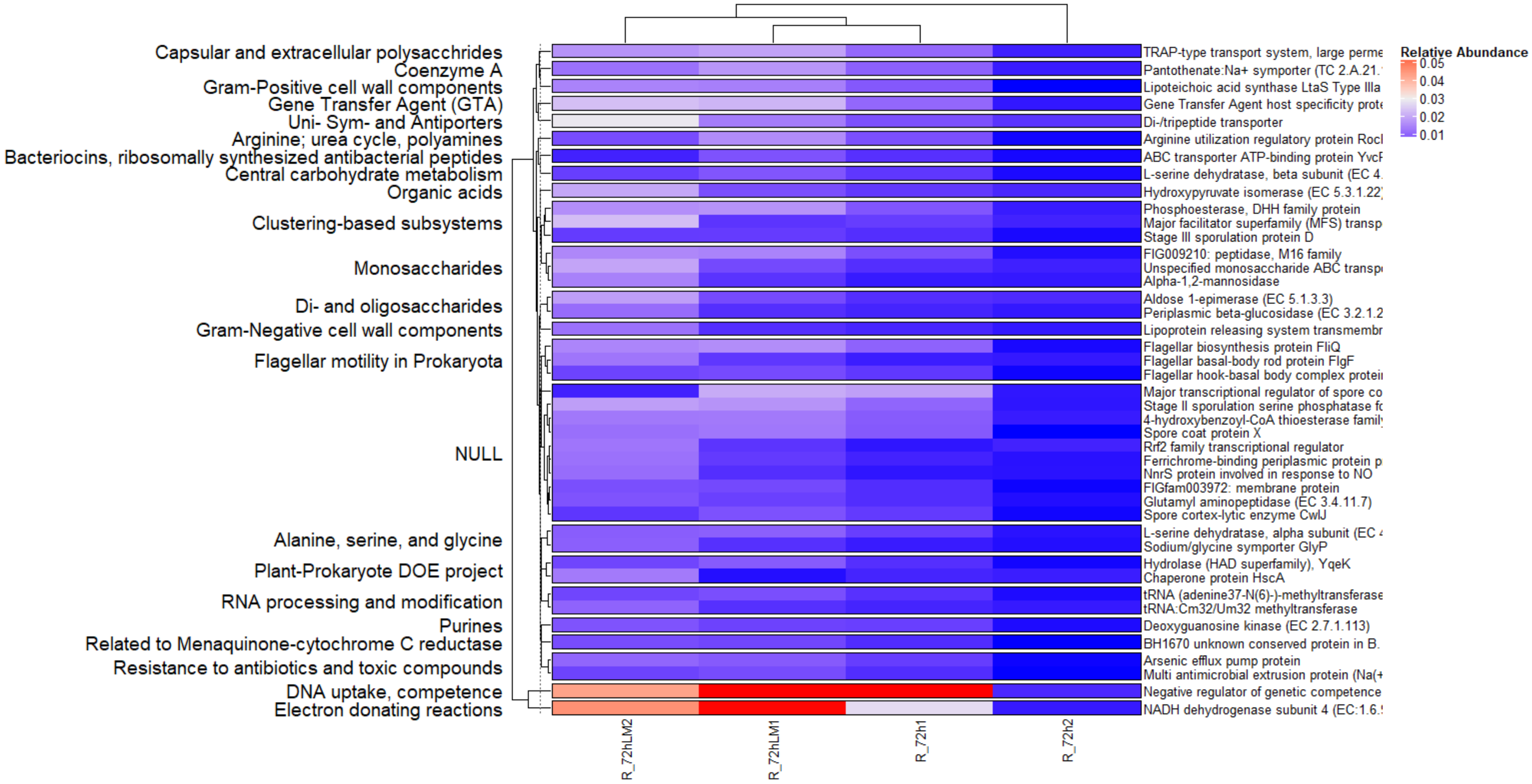

Figure S6 Experimental design for 16S rRNA gene, metagenomic, and metatranscriptomic sequencing.

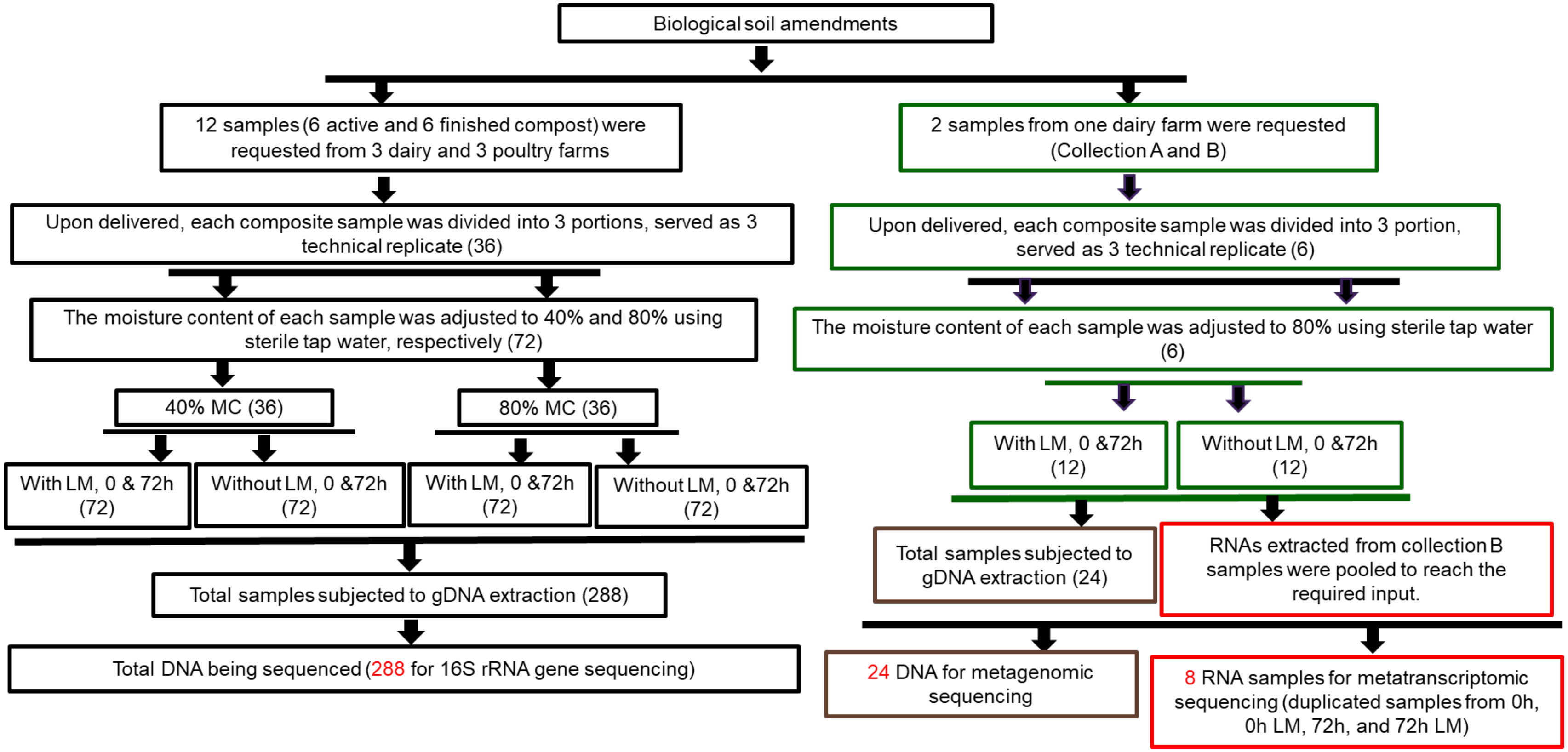

Table S1 Chemical characteristics of poultry and dairy compost samples collected from 6 farms[a]

Collection farm	Compost type	Total Nitrogen (%)	Carbon (%)	C:N (%)	Organic matter (%)	Moisture content (%)	EC (soluble salts; mmhos/cm)	pH
Dairy farm #1	Active	1.52±0.06d	24.43±0.91de	16.08±0.04b	46.55±0.92fg	26.74±0.06f	5.12±0.33cd	8.5±0bcd
	Finished	1.62±0.02d	22.22±1.07de	13.74±0.85cd	36.3±1.41hi	24.38±0.01f	4.94±0.21cd	8.45±0.07cde
Dairy farm #2	Active	1.48±0.03d	19.57±0.62e	15±0.17bc	40.7±0.17gh	53.5±0.84b	6.84±0.48c	8.45±0.07cde
	Finished	1.72±0.03cb	19.57±0.62e	11.34±0.17ef	35.95±3.18hi	47.56±3.53c	6.94±0.48c	7.85±0.07f
Dairy farm #3	Active	2.47±0.11bc	72.4±4.93a	29.35±0.76a	86.78±0.04a	83.08±1.38a	7.11±0.71c	8.8±0.14a
	Finished	3.21±0.66ab	52.94±10.27b	16.53±0.25b	77.27±0.81b	81.77±3.63a	5.32±0.86cd	8.65±0.07abcd
Poultry farm #1	Active	1.64±0.02d	27.77±1.12cde	16.97±0.45b	51.95±2.76ef	39.71±1.15d	10.47±0.46b	8.20±0e
	Finished	1.85±0.13cd	28.525±1.31cde	15.41±0.36bc	60.15±4.88de	56.67±0.16b	6.18±0.25cd	6.45±0.07g
Poultry farm #2	Active	3.13±0.07ab	38.37±0.69c	12.27±0.49de	73.45±0.64bc	25.68±0.06f	19.20±2.27a	8.70±0ab
	Finished	3.50±0.01a	34.19±1.55cd	9.77±0.47f	65.10±2.83cd	22.95±0.09f	18.88±0.35a	8.75±0.07ab
Poultry farm #3	Active	1.78±0.07cd	21.36±0.71de	12.02±0.9de	34.42±0.25hi	26.9±0.01f	5.24±0.03cd	8.75±0.07ab
	Finished	1.59±0.1d	17.55±2.13e	11.01±0.65ef	27.19±3.05i	32.54±1.51e	3.62±0.09d	8.40±0de

[a]Chemical characteristics were calculated by dry weight. Data are expressed as mean ± standard deviation (SD). Means with different letters in the same column are significantly different ($P < 0.05$).

Table S2 Microbiological analysis of poultry and dairy compost samples collected from 6 farms

Farms	Compost type	Plate count (log CFU/g) [a]					
		Total aerobic bacteria	Heterotrophs	Thermophiles	*Enterobacteriaceae*	Yeast/Mold	Actinomycetes
Dairy #1	Active	9.7±0.1a[b]	7.4±0.1d	8.3±0.1ab	4.3±0.0d	3.1±0.1c	7.5±0.1b
	Finished	7.8±0.1d	7.0±0d	7.7±0.1b	< 2.1±0e [c]	< 2.1±0d	6.7±0d
Dairy #2	Active	9.5±0a	8.9±0a	7.2±0.1bc	2.3±0e	3.1±0.1c	8.7±0a
	Finished	8.7±0c	8.6±0.1ab	6.6±0.0d	5.1±0c	< 2.3±0d	7.6±0.1b
Dairy #3	Active	9.2±0ab	8.3±0b	8.6±0.0a	6.1±0a	< 2.2±0d	7.7±0.1b
	Finished	8.5±0c	7.9±0c	7.4±0.1b	6.1±0.1a	< 2.2±0d	7.6±0.1b
Poultry #1	Active	9.0±0b	8.4±0.1b	7.5±0.1b	5.7±0.1b	5.6±0b	6.9±0.1d
	Finished	7.8±0.1d	7.7±0.1c	7.1±0.1c	5.7±0b	6.3±0.1a	7.2±0.1c
Poultry #2	Active	8.3±0.1c	7.1±0d	5.4±0.0e	< 2.1±0e	3.3±0.1c	8.3±0.1ab
	Finished	6.8±0.1e	5.6±0e	3.3±0.0f	2.2±0.1e	< 2.1±0	5.7±0.1e
Poultry #3	Active	8.1±0.1c	7.2±0d	7.4±0.0b	4.9±0.0c	< 2.5±0.1d	7.4±0.1b
	Finished	7.6±0d	7.7±0.2c	6.5±0.2d	4.9±0.1c	4.3±0c	7.3±0bc

[a] Bacterial population was calculated to log CFU/g based on dry weight of compost.

[b] Data were expressed as means ± standard deviations of triplicates samples. Means with the different lower letters in the same column are significantly different ($P < 0.05$).

[c] Detection limit for direct plating count was calculated based on the moisture content of each compost sample.

Table S3 16S rRNA gene sequencing read processing table for each compost type[a]

Compost source	Composting stage	Rawseqs (R1+R2)	Trimmed_seqs (R1+R2)	Chimera_free_seqs	Unique_seqs	Seqs (after_size_filtration)
Dairy	Active	152352±44071	145697±42645	64500±19380	566±183	63806±19222
	Finished	123430±28550	117070±27415	52887±12454	701±259	51765±12317
Poultry	Active	148585±47183	142117±45784	64378±20498	568±225	63701±20518
	Finished	104849±26862	100250±26178	43543±11393	658±379	42410±11474

[a] Data are expressed as mean ± standard deviation (SD) of sequencing reads from each group.
